# Influence of mismatched and bulged nucleotides on SNP-preferential RNase H cleavage of RNA-antisense gapmer heteroduplexes

**DOI:** 10.1038/s41598-017-12844-z

**Published:** 2017-10-02

**Authors:** Dorota Magner, Ewa Biala, Jolanta Lisowiec-Wachnicka, Ryszard Kierzek

**Affiliations:** Institute of Bioorganic Chemistry Polish Academy of Sciences, 61-704 Poznan, Noskowskiego 12/14 Poland

## Abstract

This study focused on determining design rules for gapmer-type antisense oligonucleotides (ASOs), that can differentiate cleavability of two SNP variants of RNA in the presence of ribonuclease H based on the mismatch type and position in the heteroduplex. We describe the influence of structural motifs formed by several arrangements of multiple mismatches (various types of mismatches and their position within the ASO/target RNA duplex) on RNase H cleavage selectivity of five different SNP types. The targets were mRNA fragments of *APP*, *SCA3*, *SNCA* and *SOD1* genes, carrying C-to-G, G-to-C, G-to-A, A-to-G and C-to-U substitutions. The results show that certain arrangements of mismatches enhance discrimination between wild type and mutant SNP alleles of RNA *in vitro* as well as in *HeLa* cells. Among the over 120 gapmers tested, we found two gapmers that caused preferential degradation of the mutant allele *APP* 692 G and one that led to preferential cleavage of the mutant SNCA 53 A allele, both *in vitro* and in cells. However, several gapmers promoted selective cleavage of mRNA mutant alleles in *in vitro* experiments only.

## Introduction

For more than a decade, allele-selective approaches using antisense technologies have been explored as a promising way to eliminate pathogenic alleles to treat various genetic disorders. This type of treatment may be achieved at the RNA level by enzymatic degradation of mutated mRNA by specific ribonucleases. The best targets for such approaches are genes that act in a dominant manner and present heterozygosity, meaning that in addition to the mutant allele there is also wild type one that is masked until the expression of the dominant mutant allele is repressed^1,2^. This situation is present in many neurodegenerative diseases, among which Huntington’s disease and different types of spinocerebellar ataxia that result from expanded trinucleotide repeats are the most studied targets^3–9^. The bases for distinguishing between wild type and mutant alleles are primarily SNPs (single nucleotide polymorphisms) or the length of trinucleotide repeats. In the majority of cases, SNPs are not the primary cause of disease, but variants correlate with the occurrence of wild type and mutant alleles. In turn, when targeting expanded trinucleotide repeats, the chance of antisense oligonucleotide binding is increased due to multiplication of the target sequence. However, depending on the targeted number of repeats, ASOs may be too short to directly distinguish alleles, and quantitative differentiation of alleles results from an increased frequency of binding of the oligonucleotide tools to the expanded target. Moreover, some RNAs containing expanded trinucleotide repeats are susceptible to forming hairpin structures^10^, which may be less accessible to binding by ASOs than single-stranded regions. Alleles that differ by small deletions or insertions may also be used for this purpose^5,11^, but in general, their occurrence in correlation with the target genes seems to be less frequent.

Currently, after cardiovascular diseases and cancer, neurodegenerative disorders are one of the major diseases afflicting humans. An increase in the frequency of their occurrence is associated with aging in human populations. Neurodegeneration is a complex, progressive and irreversible process of nerve cell deterioration, eventually leading to cell death. In the majority, mature neurons do not undergo cell division, which results in a strong limitation of their ability to regenerate. The accumulation of mutations, both sporadic and inherited, leads to impaired biochemical functions of many proteins in the nervous system, resulting in aggregation and formation of insoluble, toxic deposits. These pathomorphological changes in brain tissue are common in many neurodegenerative diseases, each of them involving different proteins^12,13^.

Antisense strategies provide several nucleic acid tools for RNA degradation in the context of gene silencing. Among these, the most commonly used are antisense oligonucleotides and RNAi reagents. ASOs recruit cellular RNase H1 to cleave RNA duplexed with DNA. At least five successive unmodified nucleotides at the 2′ position are required for nucleolytic activity of the enzyme^14,15^. RNA interference is an evolutionarily conserved process to repress target genes in a sequence-specific manner in a response to the presence of dsRNA molecules^16^. Small interfering RNAs (siRNAs) are agents that may be designed to induce RNAi pathways. Their presence in the cell cytoplasm induces assembly of the RISC-complex, in which they mediate cleavage of complementary mRNA targets by the Argonaute-2 (Ago2) protein^17^. The activity and specificity of RNA degradation by ASO and siRNA is increased if the constructs contain chemically modified nucleotides^5–7,18–22^.

Although antisense oligonucleotides have been known for some time, RNAi discovery has led to the rapid development of allele-selective approaches. Nevertheless, despite having a less specific mechanism of RNA degradation, antisense oligonucleotides remain an attractive tool for gene silencing. Through a wide range of novel chemical modifications of nucleotides, the specificity and selectivity ASOs can be considerably improved^9,19,21–25^. Targeting SNPs by ASOs to distinguish between wild type and mutant alleles is based on the occurrence of a single mismatch in one of the two RNA/ASO duplexes. Differentiation between the cleavage rates of these duplexes by RNase H might vary depending on the mismatch type and position with the ASO/target RNA duplex^26^. Single mismatch discrimination cleavage of a target RNA with RNase H was reported by Giles *et al*., who used chimeric methylphosphonodiester/phosphodiester oligonucleotides^27^. Later, it was shown that the sequence and structure of DNA/RNA heteroduplexes affect RNase H cleavage sites and rates^28,29^. It was found that a single mismatch results in a several-fold decrease in RNase H cleavage, and three and more mismatches completely abolished RNase H cleavage^9,30–32^.

**Figure 1:**
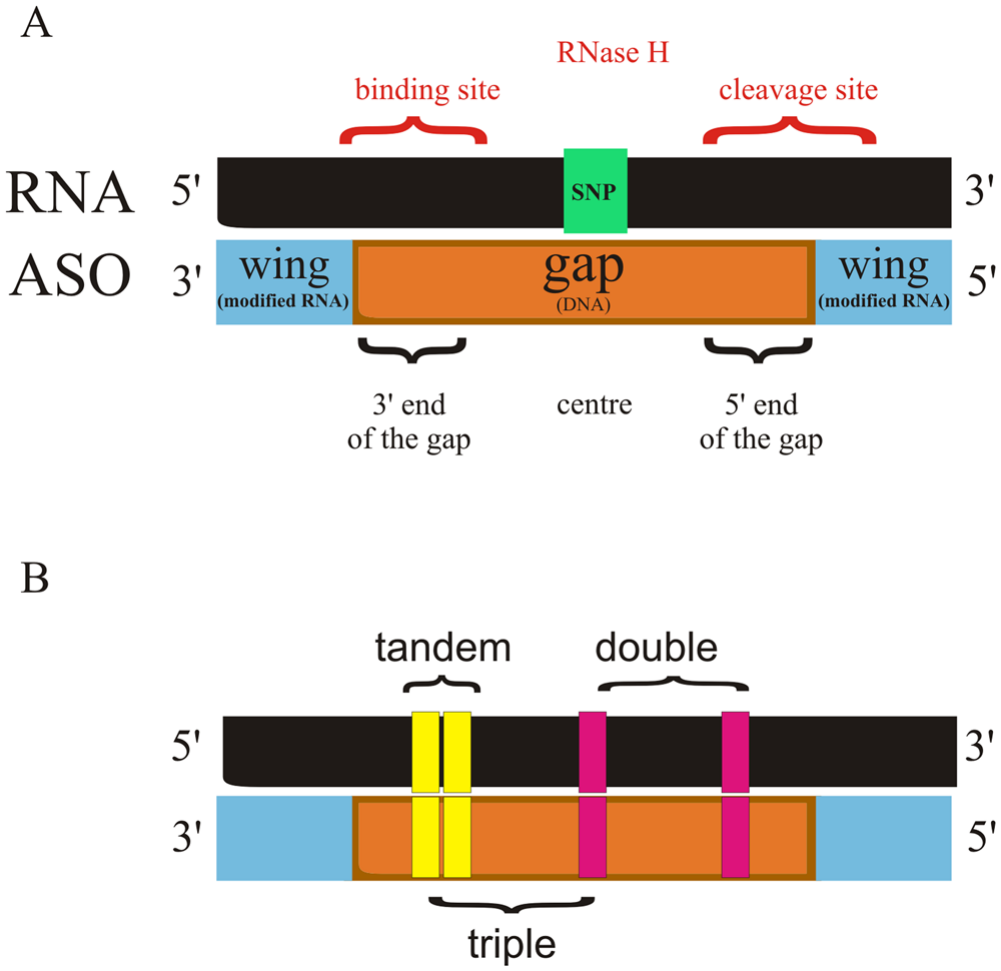
A map of RNA/gapmer heteroduplex. (**A**) General SNP positioning in the context of a gapmer structure. (**B**) Different arrangements of mismatches applied in the study.

In this study, we present the influence of several structural motifs of the RNA/ASO duplex, including bulges and different arrangements of multiplied mismatches (single, tandem, double and triple mismatches, Fig. 1B) on RNase H allele-preferential RNA cleavage *in vitro* and in *HeLa* cells. The five most common SNP types in the human genome^33–35^, occurring in *APP*, *SNCA*, *SOD1* and *SCA3* genes were chosen as the targets of this research. These SNPs, except for *SCA3*, are direct causes of rare versions of Alzheimer’s disease^36–40^, Parkinson’s disease^41,42^ and Amyotrophic Lateral Sclerosis (ALS)^43,44^. The G-to-C SNP of the *SCA3* gene (rs12895357) is commonly targeted as being associated with the expanded trinucleotide repeat allele^45,46^. By investigating this approach on different SNPs placed in model RNAs, we aimed to define the rules regarding the influence of mismatches on RNase H allele-selective cleavage of RNA within DNA/RNA duplexes with modified antisense gapmers.

*In vitro* assays were done with commercially available RNase HI from *E*. *coli*, but not with human RNase H1. *E*. *coli* ribonuclease HI is a protein comprised of 155 amino acids, consisting of a catalytic domain only, which is highly conserved compared to other RNase H1 proteins^47–49^. Human enzyme consists of 260 amino acids and is localized in the nucleus and in the mitochondria^50^. It contains additional, N-terminal RNA/DNA hybrid binding domain (HBD) that enhances processivity of the enzyme, and flexible spacer region of ~60 amino acid residues that connects it with catalytic, C-terminal domain^51,52^. Despite only 34% sequence identity, human and *E*. *coli* ribonucleases H share common catalytic mechanism and their 3D structures exhibit remarkably similarity^53^. Unlike *B*.*halodurans* enzyme and HIV-1 reverse transcriptase subunit, *E*. *coli* and human RNases H both contain a basic protrusion motif responsible for magnesium-dependent catalytic activity, deleting of which markedly reduces binding affinity for RNA/DNA hybrids^51^. The presence of additional hybrid binding domain in human RNase H1 influences precise positioning of the substrate within the active site resulting from a significantly tighter binding affinity. Thus, compared to *E*. *coli*, human enzyme shows reduced ability to move on the substrate, making the human RNase H1 cleavage more precise and focusing it between 7–10 nucleotides from the 3′-DNA/5′-RNA terminus^15^. Keeping in mind the length and composition of gapmers applied, results of *E*. *coli* RNase HI assay in this study showed that cleavage sites overlap within those positions of heteroduplex, in which cleavage by human RNase H1 is predicted.

## Results and Discussion

This study aimed to determine the parameters for designing oligonucleotides that can preferentially differentiate cleavage of two SNP variants of RNA (WT and Mut RNA) by ribonuclease H. Several arrangements of mismatches within each RNA/ASO duplex were designed to improve discrimination of single nucleotides between RNA alleles by RNase H (Fig. 1B). The presence of non-canonical base pairs decreased the thermodynamic stability of the duplexes and affected RNase HI cleavage depending on the mismatch type and its position within ASO/target RNA duplex. Initially, for most targets, the SNP was placed at the center of the RNA (position 7 of the 13 nucleotide-long gapmer). Additional mismatches were placed at various positions along the DNA gap, i.e., at the 5′-end, in the center or at the 3′-end (Fig. 1A). The gapmers were designed to minimize the number of mismatches because each additional mismatch also appears in the Mut RNA/ASO duplex.

In total, over 120 antisense oligonucleotides were tested to cause allele-preferential cleavage of Mut RNA of the *APP*, *SNCA*, *SOD1* and *SCA3* genes. Initially, the selection of ASO was based on *in vitro* RNase HI assays. For gapmers that induced selective degradation of mutant RNA, thermodynamic parameters for both RNA/ASO duplexes (wild type and mutant) were determined, and ASO gapmer transfection of *HeLa* cells was performed. The results showed that the structure of the heteroduplex, which was perturbed by mismatches, affects RNase HI cleavage yield, pattern or both, and the mismatch position may determine discrimination between RNA single nucleotide substitutions. We supposed that the SNP type may have a strong influence on the level of differentiation between alleles, because each mismatch resulted in different conformational changes to the helix, which are often dependent on the adjacent base pairs. The results supported our conjecture in this regard.

### Familial Alzheimer’s disease - *APP* mRNA point mutations as targets

Three dominant point mutations in *APP* gene occurring in exon 17, which encodes amyloid β fragment of APP protein, were chosen as targets. They are all missense mutations that increase production of amyloid β, a product of transmembrane cleavage of APP by β- and γ-secretases, the deposition of which outside neurons is a main cause of Alzheimer’s disease.

#### *APP* Flemish variant (A692G)

The *APP* Flemish variant results from a C-to-G nucleotide transversion and leads to upregulation of the secretion of both Aβ40 (less amyloidogenic) and Aβ42 (more amyloidogenic) forms of β-amyloid. This point mutation increases the solubility of Aβ peptides and the stability of peptide oligomers^54^. Conformational changes in the peptide induced by this transversion facilitate peptide adherence to the vascular endothelium, creating nidi for amyloid growth in the blood vessels of the central nervous system, and as such it is also linked to cerebral amyloid angiopathy (*CAA*)^37^.

**Figure 2:**
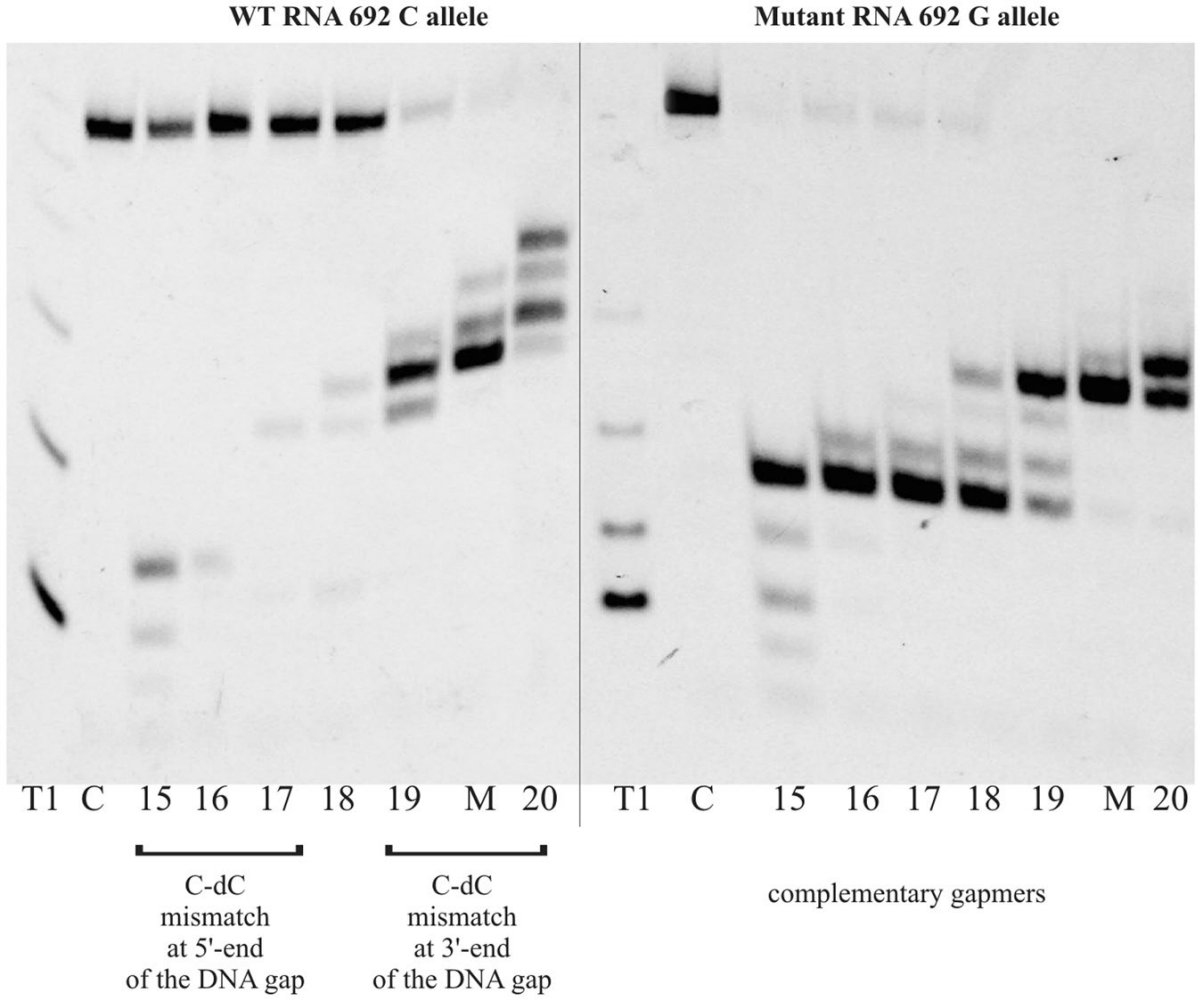
The influence of C-C mismatch position on yield of RNase H cleavage of RNA 692. T1 – RNase T1 ladder, C- control RNA (without ASO), 15–20 – gapmers b15-b20, M- gapmer bKM complementary to mutant RNA 692, order of the gapmers on gel corresponds with changing C-dC mismatch position along the gap. b15 – C-dC at position 3/13 from 5′ end of the gapmer, b20 – C-dC at position 10/13 from 5′ end of the gapmer. This gel image was cropped and rearranged to present data of interest. Original gel image is included in Supplementary Materials as Fig. S14.

**Figure 3:**
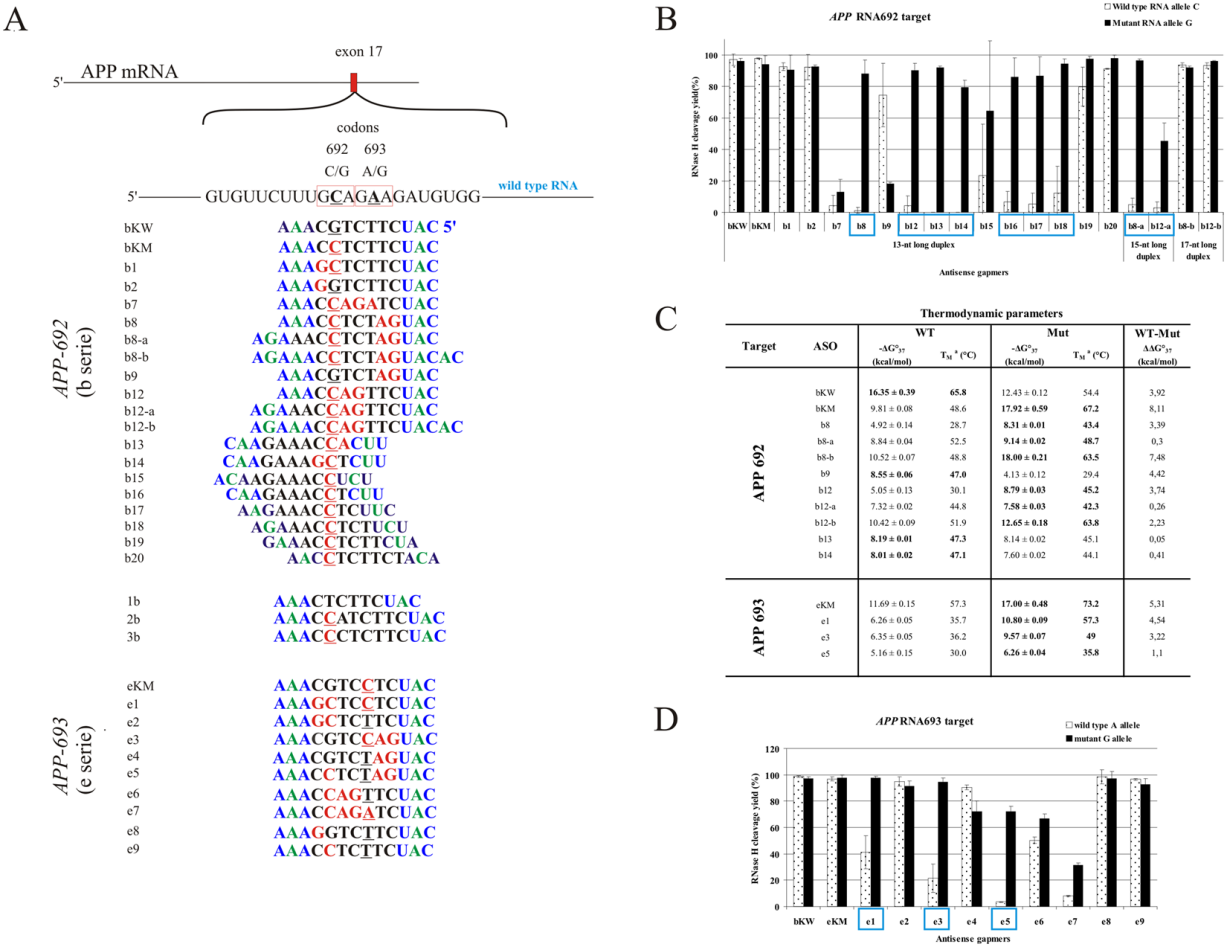
Comprehensive results of *in vitro* activity of gapmers designed to APP RNA 692 and RNA 693 targets. (**A**) Target APP mRNA sequence containing sites of C-to-G (RNA692) and A-to-G (RNA693) SNPs and designed to them antisense gapmers. SNPs in RNA strand are bolded and underlined, codons in which they occur are marked by red frame. Modified nucleotides within gapmers are marked by colours: blue-LNA, green-2′-O-MeRNA, bolded black- DNA, red-mismatched nucleotides, underlined-SNP site. (**B**) RNase H *in vitro* assay results for APP RNA692 target, showing the influence of arrangements of mismatched nucleotides within 13, 15 and 17 nt-long RNA/ASO duplexes on RNase H cleavage. Blue frames mark gapmers which cause selective degradation of Mut RNA. (**C**) Thermodynamic parameters of wild type and mutant RNA/gapmer duplexes containing mismatches. Oligonucleotides, which differentiated two alleles cleavage yields in RNase H assay were only measured. Parameters for more stable duplex of the two (wild type/ASO or mutant/ASO) are bolded. (**D**) RNase H *in vitro* assay results for APP RNA693 target, showing the influence of arrangements of mismatched nucleotides within 13 nt-long RNA/ASO duplexes on RNase H cleavage. Blue frames mark gapmers which cause selective degradation of Mut RNA.

The change of cytosine to guanine in target RNA 692 results in a C-dC mismatch in the duplex of wild type RNA and a mutant-complementary ASO. The C-dC mismatch strongly destabilizes duplexes^55,56^, but it was not known whether it will alter the activity of RNase H toward the wild type allele. *In vitro* RNase HI assays showed that the mismatch position is important for RNA cleavage yields. A single C-dC mismatch that was formed in wild type RNA/ASO duplexes caused strong thermodynamic destabilization of the helix and affected RNase HI cleavage yield of RNA 692, depending on the position within the duplex (Fig. 2). By adding subsequent mismatches next to the C-dC, tandem and triple mismatches and symmetric three-nucleotide internal loops were formed in the duplex (Fig. 3A). They increased differentiation between duplexes of wild type and mutant RNA with ASO and affected RNase HI cleavage yield much more than a single mismatch. Depending on the position of the structural motif within the duplex, diverse effects on RNase HI activity were observed (Fig. 3B). *In vitro* RNase HI assay showed highly selective cleavage of Mut RNA 692 in duplex with nine antisense gapmers (b8, b8-a, b12, b12-a b13, b14, b16, b17 and b18). All these ASO gapmers introduce single or tandem mismatches at the site of RNase H cleavage of the duplex (at the 5′-end of the DNA gap) or in the center of the duplex. However, the presence of a C-dC mismatch determines selective inhibition of cleavage of WT RNA. When tandem purine mismatches occur at the site of cleavage by RNase H (AG-dAdG, WT RNA-b9 gapmer and Mut RNA-b8 gapmer), mutant as well as wild type 692 RNA are cleaved with equal efficiency. The presence of a C-dC mismatch as a third mismatch (b8, b8-a and b12 gapmers), as the second mismatch of a tandem mismatch at the cleavage site (b13 and b14 gapmers) or even as a single mismatch at the cleavage site (b16, b17 and b18 gapmers), abolishes RNase HI activity. Additionally, a G-dG mismatch as the third mismatch in heteroduplex reduces RNase HI cleavage efficiency (Mut RNA-b9 gapmer). A triple mismatch containing C-dC forms a small internal loop that, when placed in the center of the duplex, inhibits RNase HI cleavage of WT RNA (b12 gapmer). However, WT RNA cleavage is half as efficient when the C-dC mismatch is separated from the tandem mismatch by a few nucleotides (b8 gapmer). When the C-dC mismatch was placed in a tandem mismatch at the 3′-end of the DNA gap (that includes the RNase H binding site of the duplex; gapmers b1 and b2), no difference in cleavage yield of RNase HI between two RNA SNP variants was observed.

The effectiveness of the triple-mismatch motifs described above introduced to 13 nucleotide WT or Mut RNA/ASO duplexes was also confirmed for 15 and 17 nucleotide-long gapmers, which were marked with the suffix “–a” and “–b,” respectively. In the case of 15-mer gapmers, the gap region was elongated from 7 to 9 nucleotides, and the selective Mut RNA cleavage effect was maintained. In the case of 17-mer gapmers, the flanking region was elongated from three to five nucleotides on both sides, which made the resulting RNA/ASO duplex more rigid and minimized the destabilizing effect of the three mismatches. As a consequence, selective degradation of Mut RNA by RNase HI was abolished, which confirmed the presumption that disruption of the helical structure at the cleavage site of the duplex may lower RNase H activity.

**Figure 4:**
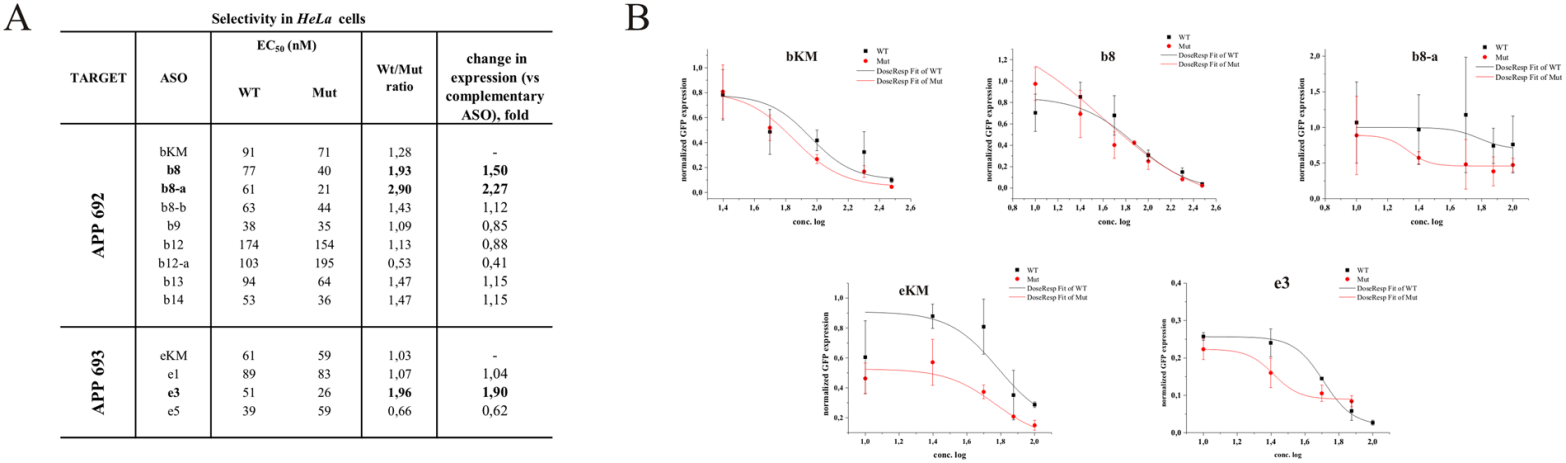
Activity of selected gapmers, designed to APP RNA 692 and RNA 693 targets, in *HeLa* cells. (**A**) Results of ASOs transfection to *HeLa* cells. The EC_50_ values for gapmers tested in presence of WT and mutant alleles were estimated based on dose-response curves fitted to experimental data in Origin Lab 8.0 software (**B**). The data were gathered from at least three separate experiments involving five concentrations in the range 10–100 nM. Comparison of EC_50_ values for gapmers between WT and Mutant target RNA alleles allowed to evaluate allele-preference for RNA cleavage of tested gapmers. Two last columns of the table present WT/Mut ratio of gapmer selectivity and the selectivity of mismatched gapmers relative to complementary gapmer. (**B**) Dose-response curves for selected antisense gapmers. Upper charts – ASO targeting APP Flemish variant (RNA692): bKM - referenced ASO gapmer (mutant complementary to RNA692 target); b8, b8-a – gapmers presenting selectivity to Mut RNA692 in *HeLa* cells; Lower charts – ASO targeting APP Arctic variant (RNA693) eKM - referenced ASO gapmer (mutant complementary to RNA693 target); e3 – gapmer selective to Mut RNA693 target in *HeLa* cells; for statistics see Supplementary data.

Among the 20 antisense gapmers designed to induce selective cleavage of mutant G variant of RNA 692, eight (b8, b8-a, b12, b12-a, b13, b14, b16 and b17) were selected in RNase HI assays *in vitro*, because they reduced the level of mutant RNA at least two-fold without affecting WT RNA (Fig. 3A). These eight gapmers, and a few more with interesting activity *in vitro* (for example they differentiated cleavage in the opposite way, such as the b9 gapmer), were cotransfected into *HeLa* cells at five different concentrations in the range of 0–150 nM ASO together with plasmids carrying an over one-hundred nucleotide fragment of the *APP* gene with the C-to-G substitution. To compare the allele-selective effect of the gapmers, EC_50_ values were determined for each RNA variant-gapmer pair (Fig. 4A). Under cellular conditions, only two ASOs retained the allele-preferential action observed *in vitro*. The highest selectivity of degradation toward Mut RNA was observed for the b8-a gapmer, which resulted in an almost three-fold excess in the amount of WT RNA compared to mutant RNA. Furthermore, b8-a showed more than twice as much preference toward cleavage of the Mut RNA than the mutant-complementary bKM gapmer, and 1.5-fold stronger preference than the primary b8 gapmer (Fig. 3E).

An interesting observation was made in *HeLa* cells concerning selectivity of gapmer action depending on the pattern of gapmer elongation. A comparison of the WT and Mut RNA ratios obtained after *HeLa* treatment with b8 and b12 gapmers and their elongated versions (b8-a, b12-a, b8-b, b12-b) demonstrated more preferential cleavage of Mut RNA with b8-a and b12-b. This means that in the case of separated mismatches (b8 pattern), gapmer selectivity was improved by elongation of the gap region by two nucleotides, but for coupled mismatches that occur centrally in the duplex (b12 pattern), better selectivity was observed when the flanking wings of the gapmer was elongated (Fig. 3A). In the case of the elongation of the b8 gapmer, where three mismatches occur as single C-dC and tandem AG-dGdG mismatches at the opposite ends of the DNA core of the gapmer, the selectivity of RNase H1 cleavage increased. Elongation of the DNA gap was not symmetric, but occurred at the 3′-end of the gap, so consequently, the third C-dC mismatch was shifted to the center of the heteroduplex. On the other hand, the extension of the flanking terminal wings of the b8 gapmer caused a decrease in the allele-selectivity of degradation. In the case of the b12 arrangement of mismatches, extending the 7-nucleotide DNA gap by two nucleotides (gapmer b12-a) resulted in decreased selectivity and an increased level of WT RNA. On the other hand, extension of the flanking region (gapmer b12-b) caused increase of both, WT and Mut constructs expression.

#### *APP* Arctic variant (E693G)

The Arctic variant of the *APP* gene results from an A-to-G transition in codon 693 and causes amino acid substitution of glutamic acid by glycine. This change in the APP protein increases the rate and amount of protofibrill formation and induces resistance to neprilysin, a metalloproteinase that inactivates amyloid-β by degrading it^38,57,58^. For that reason, the mutation may result in an aggressive Alzheimer’s course. Given the RNA-antisense oligonucleotide interactions, in this SNP, a destabilizing A-dC single mismatch occurs in the duplex of the WT RNA-mutant-complementary gapmer eKM. Due to the mismatch, binding of the eKM gapmer to WT RNA 693 (A allele) is about two times weaker than to the complementary mutant RNA (G allele)^26^. However, the central position of this mismatch type did not influence RNase HI selectivity. Among the few mismatch arrangements tested (Fig. 3A), three, represented by e1, e3 and e5 oligonucleotides, showed significant differentiation of degradation between WT and Mut RNAs in the *in vitro* RNase HI assay (Fig. 3D). *HeLa* cell experiments showed that only the e3 gapmer considerably lowered the level of the mutant RNA 693 compared to the wild type RNA in the tested concentration range (Fig. 4A and B). The e3 gapmer and its target RNA form a duplex containing the tandem mismatch AG-dAdG in the case of Mut RNA, and the triple mismatch AAG-dCdAdG in the case of WT RNA, located in the RNase H cleavage region of the duplex. The tandem purine mismatch does not reduce the RNase HI cleavage rate of the Mut RNA, but the third non-canonical A-dC pair clearly inhibits the cleavage of WT RNA. Similar conclusions were made and discussed above for the C-to-G transversion occurring adjacent in the same target RNA, which causes a C-dC mismatch. For these *APP* target RNA duplexes, which contain strongly destabilizing 2′-deoxycytidine mismatches at the RNase H cleavage site of the duplex, inhibition of RNA cleavage was observed. Furthermore, these observations confirm that three mismatches can differentiate *in vitro* hydrolysis of RNA SNP alleles by RNase HI in duplexes with gapmers, in most cases regardless of their position. However, the differentiation ability of single, double or tandem mismatches is less efficient and is strongly dependent on mismatch type and position.

#### *APP* London variant (V717I)

**Figure 5:**
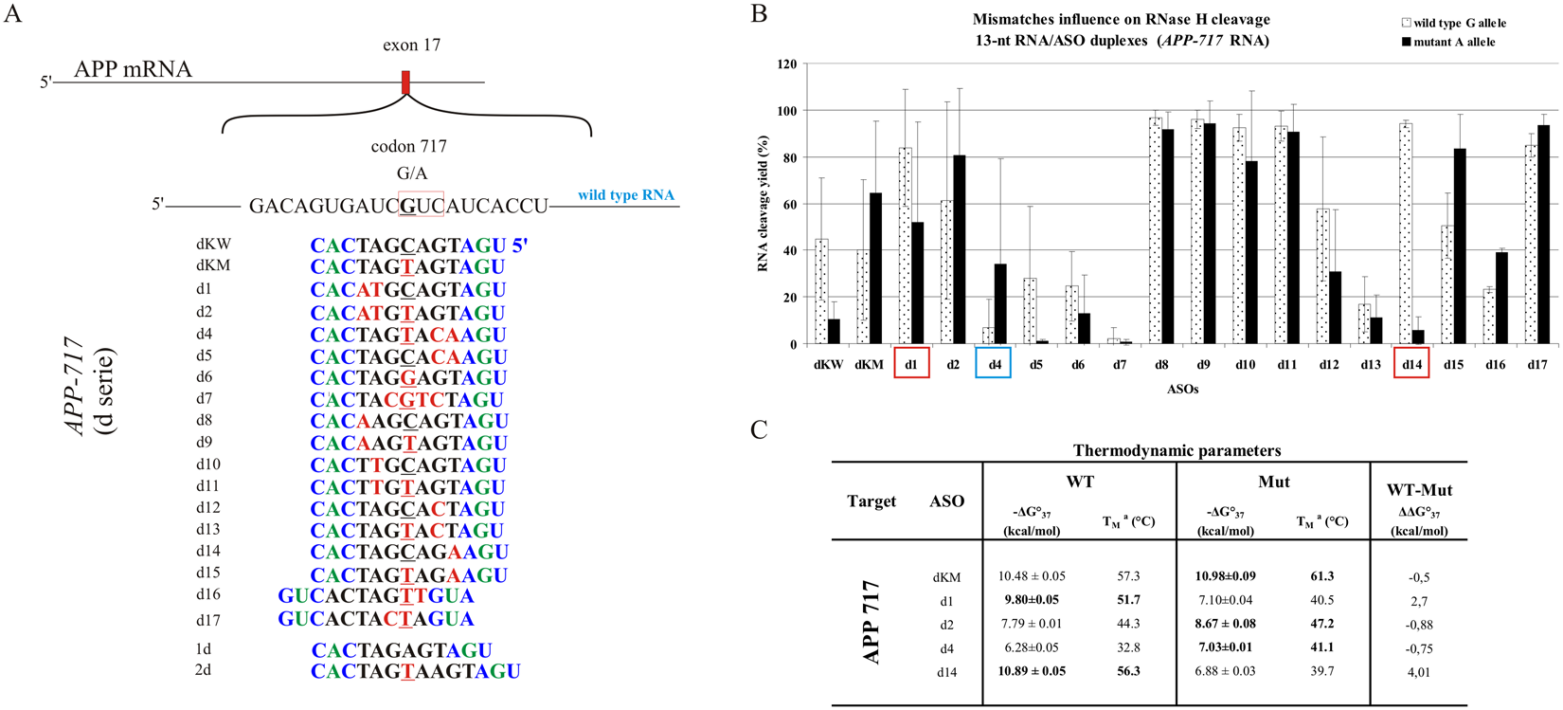
Comprehensive results of *in vitro* activity of gapmers designed to APP RNA 717 target (APP London variant). (**A**) Target APP mRNA sequence containing site of G-to-A (RNA717) SNP and designed to it antisense gapmers. SNP in RNA strand is bolded and underlined, codon in which it occurs is marked by red frame. Modified nucleotides within gapmers are marked by colours: blue-LNA, green-2′-O-MeRNA, bolded black- DNA, red-mismatched nucleotides, underlined-SNP site. (**B**) RNase H *in vitro* assay results for APP RNA717 target, showing the influence of arrangements of mismatched nucleotides in 13 nt-long RNA/ASO duplexes on RNase H cleavage. Blue frames mark gapmers which cause preferential cleavage of Mut RNA. Red frames mark gapmer which cause preferential cleavage of WT RNA. (**C**) Thermodynamic parameters of APP 717 wild type and mutant RNA/gapmer duplexes containing mismatches. Oligonucleotides, which differentiated two alleles cleavage yields in RNase H assay were only measured. Parameters for more stable duplex of the two (wild type/ASO or mutant/ASO) are bolded.

**Figure 6:**
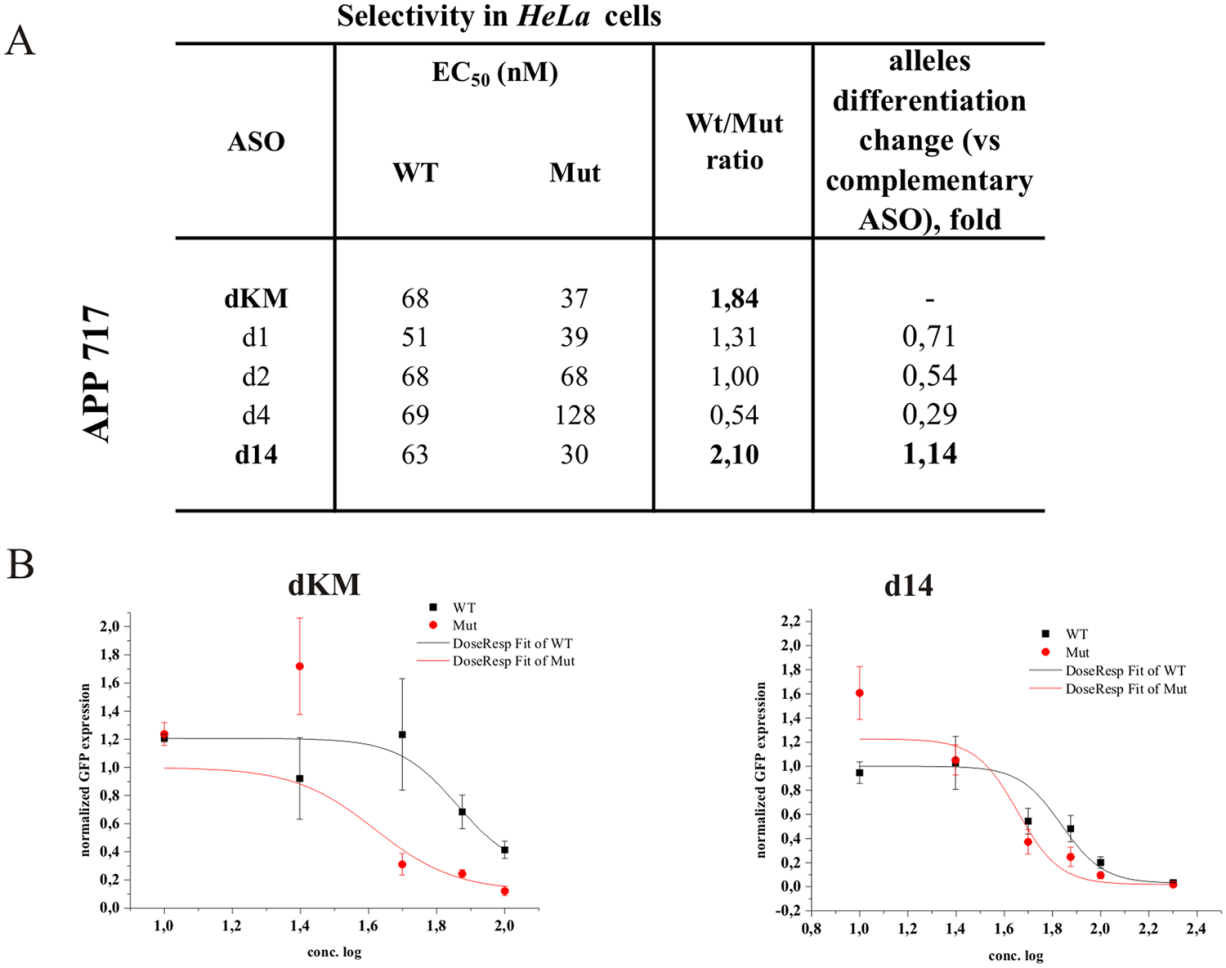
Activity of selected gapmers, designed to APP 717 target, in *HeLa* cells. (**A**) Results of ASOs transfection to *HeLa* cells. The EC_50_ values for gapmers tested in presence of WT and mutant alleles were estimated based on dose-response curves fitted to experimental data in Origin Lab 8.0 software (for statistics see Supplementary data). The data were gathered from at least three separate experiments involving five concentrations in the range 10–100nM. Comparison of EC_50_ values for gapmers between WT and Mutant target RNA alleles allowed to evaluate allele-preference for RNA cleavage of tested gapmers. Two last columns of the table present WT/Mut ratio of gapmer selectivity and the selectivity of mismatched gapmers relative to complementary gapmer dKM. Plots present dose-response curves for selected antisense gapmers. (**B**) Dose-response curves for selected antisense gapmers. dKM - referenced ASO gapmer (mutant complementary to RNA717 target); d14 – ASO gapmer presenting SNP preference in *HeLa* cells, however not in the RNase H assay *in vitro*. Although the both models for d14 gapmer were statistically insignificant (P > 0.05), validation experiment confirmed SNP-preferential cleavage in concentrations determined from the curves (Fig. S13).

Several mutations in codon 717 of the *APP* gene are well known in the context of Alzheimer’s disease, and one of the most studied of these is the G-to-A nucleotide transition. This mutation results in a change of valine to isoleucine in the amyloid-β precursor protein and causes increased secretion of the Aβ42 form, promoting amyloid plaque formation and deposition in the brain. We observed only slightly impaired stability and RNase HI cleavage yield of the WT RNA duplex in comparison to the complementary Mut RNA duplex (dKW and dKM gapmers, Fig. 5). The presence of G-dT in the WT RNA duplex in place of A-dT does not disturb the duplex structure necessary for RNase H activity. Cleavage yield of WT RNA 717 G in comparison to Mut RNA 717 A decreased only approximately 20%, and the thermodynamic stability was lowered by only 0.5 kcal/mol (Fig. 5B and C). Slightly more selective Mut RNA degradation occurred in presence of the d15 gapmer, which distinguished cleavage of WT and Mut RNA *in vitro* by 30%. However, among twenty different structural motifs that were analyzed within the region adjacent to the SNP (Fig. 5A), none of the designed mismatch arrangements definitely inhibited WT RNA cleavage while causing preferential degradation of Mut RNA. Surprisingly, the gapmer complementary to Mut RNA (dKM), carrying this subtle G-dT mismatch in the wild type duplex, presented the most effective differentiation of allele degradation in *HeLa* cells, causing an almost two-fold reduction of Mut RNA relative to WT RNA (Fig. 6). This was the best result obtained for the case of G-to-A transition. Neither double mismatches (two mismatches separated from each other by one or more base pairs), nor tandem mismatches that contained the SNP, were sufficient for preferential inhibition of RNase H1 activity towards WT RNA. Usually, the mismatches caused a significant change at the cleavage site, but did not differentiate the amount of RNA cleaved between the RNA alleles (Figure S1C).

### Further searching for a G-to-A selective antisense gapmer: SNCA point mutations linked to Parkinson’s disease

Two more G-to-A point mutations in a different gene context were also analyzed as potential targets for the SNP-preferential antisense approach. They both occur in the α-synuclein transcript and lead to different type of neurodegeneration related to development of early-onset Parkinson’s disease^41,42^. α-Synuclein is involved in the regulation of dopamine release and transport. Little is known about the direct mechanism of action of these mutations, except that they probably change the protein structure and lead to its aggregation. The first of the target G-to-A substitutions of the *SNCA* transcript leads to change of glutamic acid to lysine in codon 46 (E46K), which corresponds to amino acid 46 in the N-terminal fragment of the alpha-synuclein protein. This change results in a significant increase in α-synuclein binding to negatively charged liposomes. Moreover, it leads to increased rate and amount of filaments assembly, a step in α-synuclein aggregation that results in the formation of the brain lesions that are the hallmarks of Parkinson’s disease^59,60^. The second G-to-A transition in the *SNCA* transcript results in a change in codon 53 from alanine to threonine (A53T) and it is only approximately 20 nucleotides away from E46K. It also increases rate and amount of filaments assembly, that differ from those formed by the 46 K mutant and by wild type as well. Targeting these two G-to-A transitions was a way of checking if this type of substitution truly has no effect on RNase H cleavage, independently of the position they occupy within the RNA/gapmer duplex.

**Figure 7:**
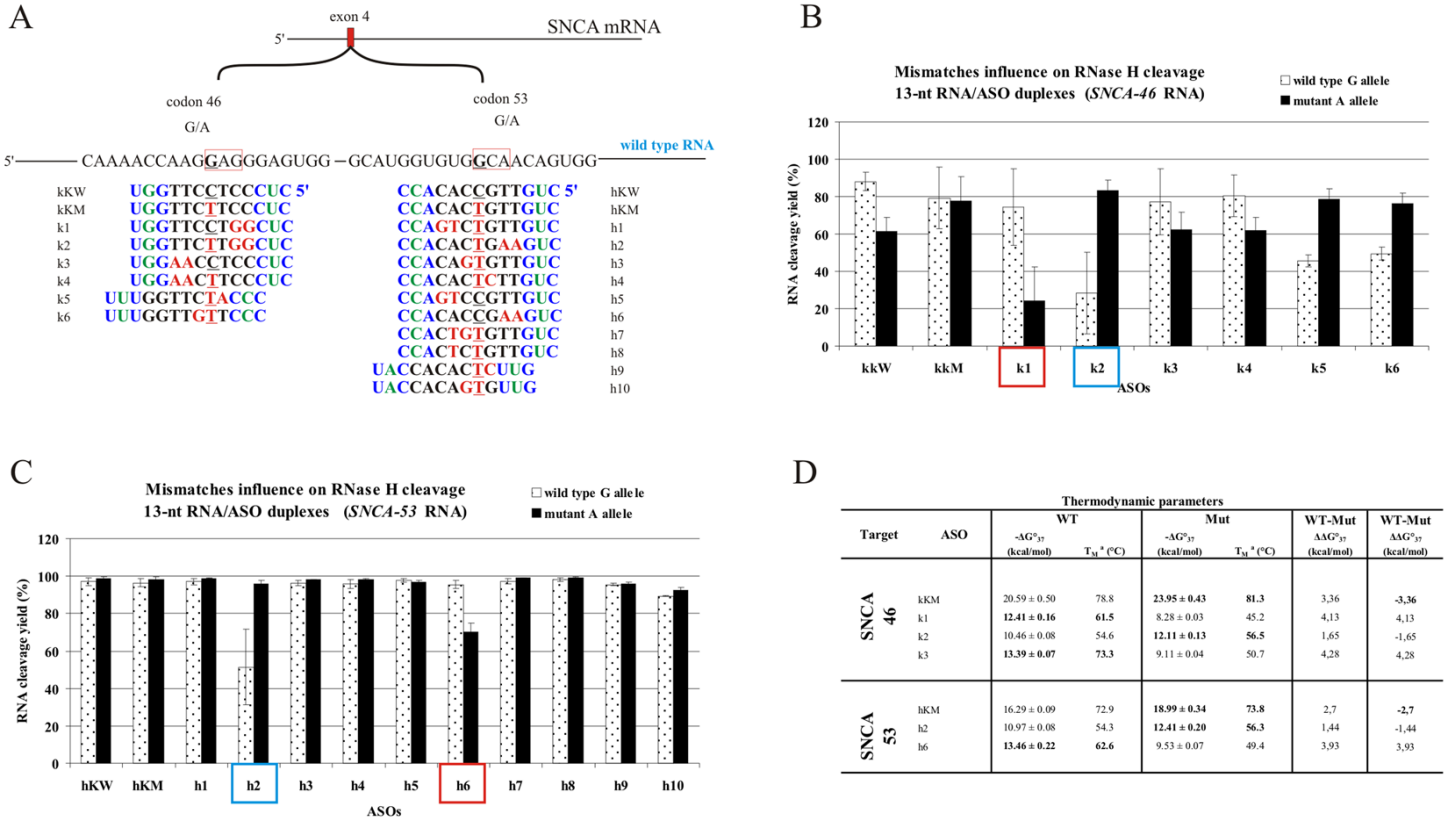
Comprehensive results of *in vitro* activity of gapmers designed to SNCA RNA 46 and RNA 53 targets. (**A**) Target SNCA mRNA sequence containing sites of two G-to-A SNPs (RNA46 and RNA53) and designed to them antisense gapmers. SNPs in RNA strand are bolded and underlined, codons in which they occur are marked by red frame. Modified nucleotides within gapmers are marked by colours: blue-LNA, green-2′-O-MeRNA, bolded black- DNA, red-mismatched nucleotides, underlined-SNP site. (**B**) RNase H *in vitro* assay results for SNCA RNA46 target, showing the influence of arrangements of mismatched nucleotides in RNA/ASO duplexes on RNase H cleavage. Blue frames mark gapmers which cause selective cleavage of Mut RNA, red frames mark gapmers which preferentially cause wild type RNA cleavage. (**C**) RNase H *in vitro* assay results for SNCA RNA53 target, showing the influence of arrangements of mismatched nucleotides in RNA/ASO duplexes on RNase H cleavage. Blue frames mark gapmers which cause selective cleavage of Mut RNA, red frames mark gapmers which preferentially cause wild type RNA cleavage. (**D**) Thermodynamic parameters of SNCA wild type and mutants RNA/gapmer duplexes containing mismatches. Oligonucleotides, which differentiated two alleles cleavage yields in RNase H assay were only measured. Parameters for more stable duplex of the two (wild type/ASO or mutant/ASO) are bolded.

The *in vitro* RNase HI assay indicated small differences in cleavage yields between the two RNA alleles. In both cases of these *SNCA* transitions, the most selective cleavage of the mutant A-allele was observed with gapmers k2 and h2, which introduced tandem mismatches in the RNase H cleavage region (two nucleotides at the 5′-end of the DNA gap), and the SNP site was placed centrally within duplex. Additionally, in both cases tandem mismatches were purine mismatches (GG-dGdG for RNA 46 with gapmer k2 and AA-dAdA for RNA 53 with gapmer h2). UV-melting experiments showed only small differences in the thermodynamic stability between duplexes of WT and Mut RNA and the gapmer (ΔΔG°_37_ of −1.65 and −1.44 kcal/mol, respectively; Fig. 7D), due to small differences in stability between the G-dT and A-dT pairs. However, relative to fully complementary gapmers, tandem purine mismatches at 5′-end of the DNA gap strongly diminish duplex thermodynamic stability (ΔΔG°_37_ between Mut RNA 46 duplexes of kKM and k2 was 11.84 kcal/mol and between Mut RNA 53 duplexes of hKM and h2 was 6.58 kcal/mol). Despite the large differences in stability between the tandem mismatch-containing (k2 and h2) and tandem mismatch-free duplexes (kKM and hKM, respectively), the efficiency of their cleavage by RNase HI is almost unaffected until the third centrally placed mismatch occurs. Its presence significantly reduces the cleavage efficiency of WT RNA, by 75% in case of RNA 46 and by 50% in case of RNA 53. Interestingly, in both cases of this transition, when the third, centrally placed mismatch occurred in Mut RNA duplexes and WT RNA duplexes contained only tandem mismatches at the 5′-end of the DNA gap, preferential cleavage of wild type RNA occurred (k1 and h6 gapmers for RNA 46 and RNA 53, respectively). This observation confirms the structural significance of this arrangement of mismatches for selective RNase HI degradation of RNA alleles.

**Figure 8:**
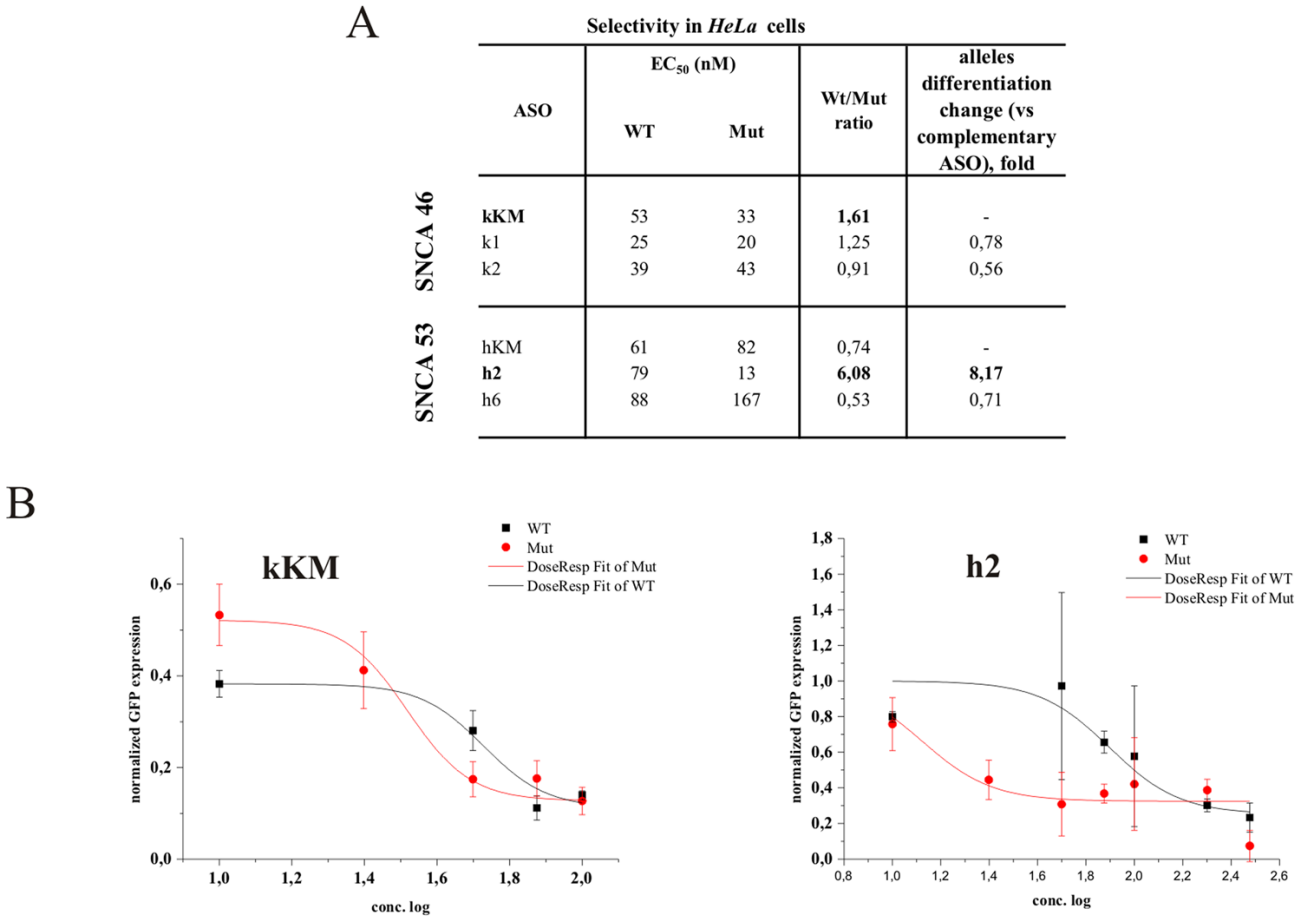
Activity of selected gapmers, designed to SNCA RNA 46 and RNA 53 targets, in *HeLa* cells. (**A**) Results of ASOs transfection to *HeLa* cells. The EC_50_ values for gapmers tested in presence of WT and mutant alleles were estimated based on dose-response curves fitted to experimental data in Origin Lab 8.0 software (for statistics see Supplementary data). The data were gathered from at least three separate experiments involving five concentrations in the range 10–100nM. Comparison of EC_50_ values for gapmers between WT and Mutant target RNA alleles allowed to evaluate allele-preference for cleavage of tested gapmers. Two last columns of the table present WT/Mut ratio of gapmer selectivity and the selectivity of mismatched gapmers relative to complementary gapmers (kKM for RNA 46 target and hKM for RNA 53 target). (**B**) Dose-response curves for antisense gapmers causing preferential cleavage of Mut 46 RNA (kKM) and Mut 53 RNA (h2).

In *HeLa* cell assays, we observed different results for each G-to-A target. The preference of the h2 gapmer toward Mut RNA 53 degradation was confirmed, demonstrating that its effective inhibitory concentration in the case of Mut RNA was over 6-fold lower than for WT RNA (Fig. 8A). The h2 gapmer was also 8-fold more selective toward mutant RNA than the complementary hKM gapmer. Furthermore, the h6 gapmer, which showed some undesirable selectivity toward WT RNA 53 degradation *in vitro*, maintained this profile in *HeLa* cells. However, for the RNA 46 target, the k2 gapmer's activity was not consistent with the *in vitro* results, remaining non-selective towards any of RNA 46 alleles. Unexpectedly, the mutant-complementary gapmer kKM showed some preferential degradation of Mut RNA 46, causing its cleavage to a concentration 1.6-fold lower than cleaved WT RNA. A similar profile of activity for a mutant-complementary antisense gapmer was observed in the case of *APP* RNA 717 G-to-A transition, in which poor gapmer selectivity *in vitro* turned out to be significant in *HeLa* cells.

### Amyotrophic Lateral Sclerosis variant related with C-to-U transition in SOD1 RNA

One of the most frequent substitutions in the human genome is the C-to-T transition, because of the major mechanism for newly occurring mutations, which is deamination of cytosine to uracil. In the context of neurodegeneration, we decided to target our SNP-selective antisense approach to the A4V mutation in the *superoxide dismutase 1* gene that corresponds to a C-to-U transition. The mutation causes Amyotrophic Lateral Sclerosis (ALS) and it is specific for the U.S. population. Approximately 50% of SOD1-ALS patients in U.S. carry the A4V substitution^43,44,61^. It acts in a dominant manner (as most of the known ALS-causing SOD1 mutations), and a single mutant copy of the gene is sufficient to cause the disease. The mechanism by which the A4V mutation causes ALS probably relies on disturbing the formation of the SOD1 homodimer, which is the active form of the protein. As a result of loss of stability of the dimer structure, SOD1 aggregation is accelerated, which causes faster progression of neuronal degeneration^62^.

**Figure 9:**
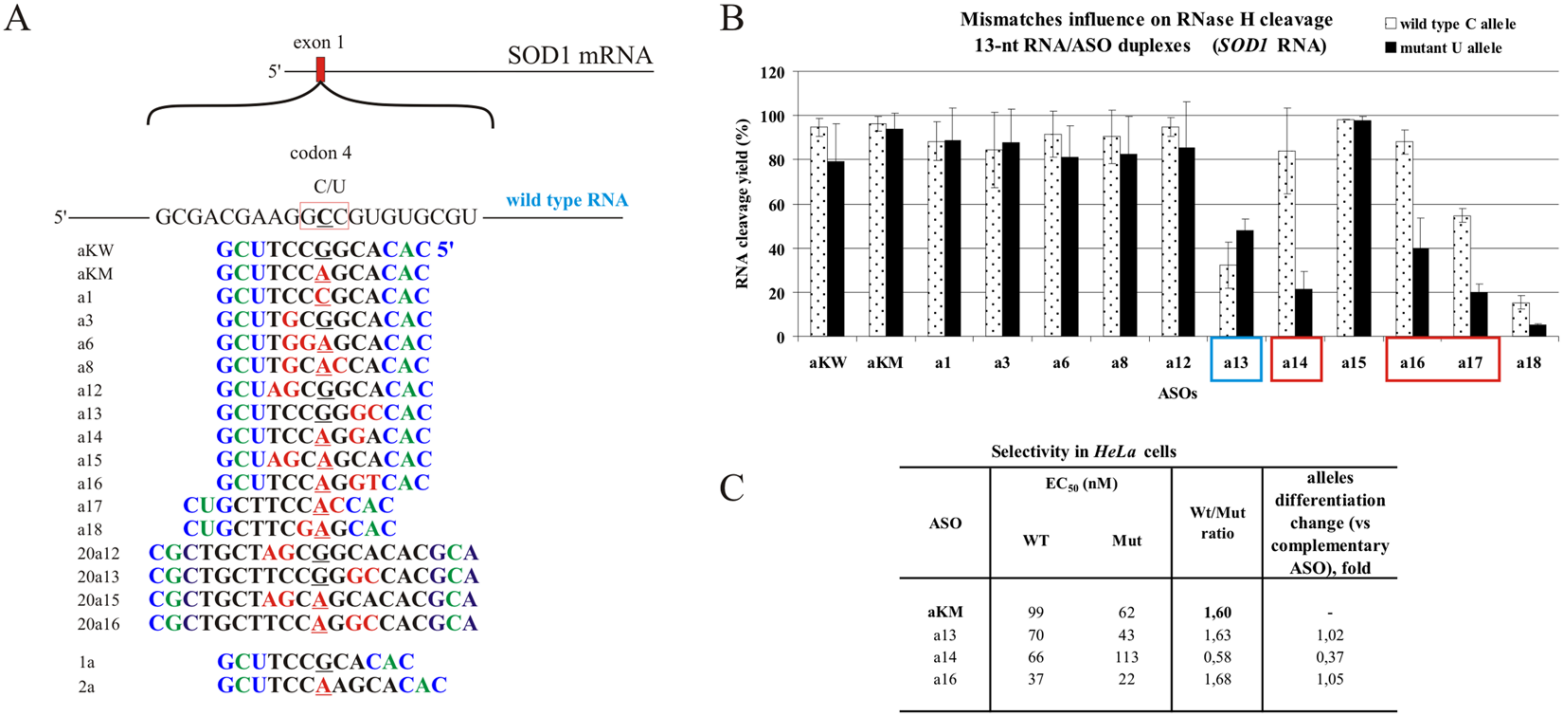
Activity of gapmers designed to SOD1 RNA 4 target *in vitro* and in *HeLa* cells. (**A**) Target SOD1 mRNA sequence containing site of C-to-U SNP (RNA4) and designed to it antisense gapmers. SNP in RNA strand is bolded and underlined, codon in which it occur is marked by red frame. Modified nucleotides within gapmers are marked by colours: blue-LNA, green-2′-O-MeRNA, bolded black- DNA, red-mismatched nucleotides, underlined-SNP site. (**B**) RNase H *in vitro* assay results for SOD1 RNA4 target, showing the influence of arrangements of mismatched nucleotides in RNA/ASO duplexes on RNase H cleavage. Red frames mark gapmers which cause undesirable effect of preferential wild type RNA cleavage. Blue frames mark gapmers which cause selective degradation of Mut RNA. (**C**) Results of ASOs transfection to *HeLa* cells. The EC_50_ values for gapmers tested in presence of WT and mutant alleles were estimated based on dose-response curves fitted to experimental data in Origin Lab 8.0 software (for statistics see Supplementary data). The data were gathered from at least three separate experiments involving five concentrations in the range 10–100nM. Comparison of EC_50_ values for gapmers between WT and Mutant target RNA alleles allowed to evaluate allele-preference for cleavage of tested gapmers. Two last columns of the table present WT/Mut ratio of gapmer selectivity and the selectivity of mismatched gapmers relative to complementary gapmer aKM.

The starting point for antisense gapmer design targeting the C-to-U transition case in the *SOD1* transcript was a single C-dA mismatch, occurring in the wild type SOD1 RNA 4 duplex. When placed in the center of the duplex, the mismatch caused moderate thermodynamic destabilization of the RNA/gapmer duplex, but did not decrease the efficiency of RNase HI cleavage of WT RNA (Figs S2A and 9B, respectively). Among all tested arrangements of mismatches (Fig. 9A) we were not able to find any that would cause selective mutant RNA 4 degradation by RNase H *in vitro*. We found very small differences in the efficiency of RNase HI cleavage of both allelic forms of SOD1 RNA. However, for three antisense gapmers: a14, a16 and a17, some preferential cleavage of WT RNA was observed (Fig. 9B). These gapmers contain mismatched nucleotides at the 5′-end of the DNA gap (the region directly involved in cleavage), and a third additional mismatch, which appears as a result of the transition, enhances *in vitro* cleavage of WT RNA 4. However, in *HeLa* cells we observed preferential wild type allele cleavage only in presence of a14 gapmer (Figs 9C and S2B). The results of RT-qPCR experiments from cells transfected with aKM, a13 and a16 gapmers demonstrated their minor preference for Mut RNA degradation, and this result confirms only the low a13 selectivity *in vitro*. Gapmer a16, which strongly discriminate wild type allele *in vitro*, were not preferential toward wild type RNA in cells. These results led us to conclude that in cellular conditions, the duplex interactions may be hindered in comparison to buffer conditions, or may be disturbed by cellular crowding and interactions with proteins. One of the rules for searching for appropriate targets for siRNAs includes avoiding sites at the 5′-end of mRNA, because this region is mostly involved in interactions with translational machinery and might not be easily accessible to oligonucleotide binding^63^. This may be the explanation for this particular case, as the C-to-U transition occurs at the fourth codon in the first exon of the *SOD 1* transcript, which therefore can be less accessible for interactions with antisense oligonucleotides.

### *SCA3* RNA: an example of G-to-C transversion segregating with expanded CAG repeats

The *SCA3* gene encodes ataxin-3, a deubiquitinating enzyme involved in protein homeostasis maintenance and degradation of misfolded chaperone substrates^64^. A CAG repeat expansion in the coding region of the protein results in Machado-Joseph disease, a type of spinocerebellar ataxia linked to the degeneration of neurons in the brainstem and spinal cord. Directly after the repeat sequence, a G-to-C SNP occurs (rs12895357, dbSNP), of which variants G and C are associated with wild type and mutant alleles, respectively^65^. We chose this SNP as a target for searching for allele-selective gapmers, because it has already been used for similar purposes by other research groups^65,66^.

**Figure 10:**
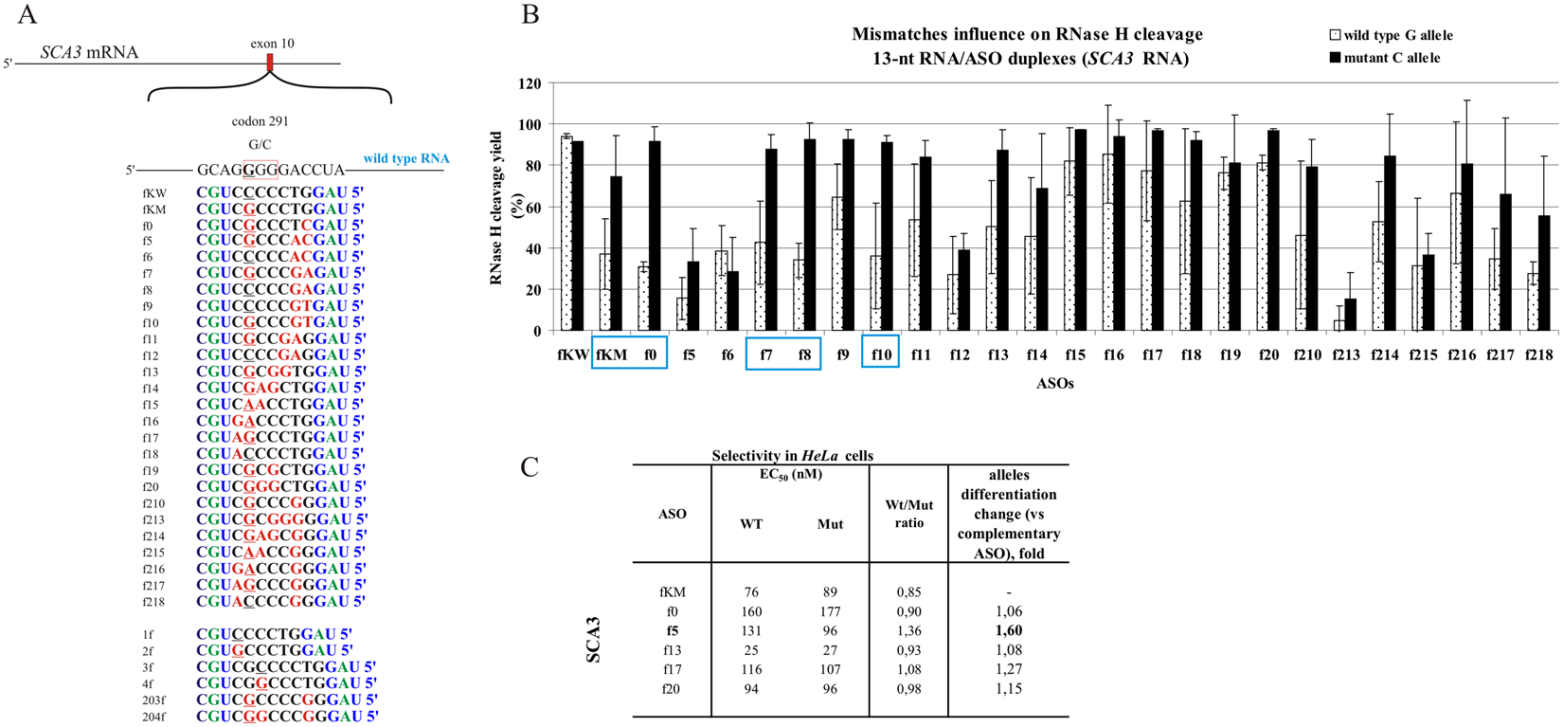
Activity of gapmers designed to SCA3 RNA target *in vitro* and in *HeLa* cells. (**A**) Target SCA3 mRNA sequence containing site of G-to-C SNP and designed to it antisense gapmers. SNP in RNA strand is bolded and underlined, codon in which it occur is marked by red frame. Modified nucleotides within gapmers are marked by colours: blue-LNA, green-2′-O-MeRNA, bolded black- DNA, red-mismatched nucleotides, underlined-SNP site. (**B**) RNase H *in vitro* assay results for SCA3 RNA target, showing the influence of arrangements of mismatched nucleotides in 13 nt-long RNA/ASO duplexes on RNase H cleavage. Blue frames mark gapmers which cause preferential cleavage of Mut RNA. (**C**) Results of ASOs transfection to *HeLa* cells. The EC_50_ values for gapmers tested in presence of WT and mutant alleles were estimated based on dose-response curves fitted to experimental data in Origin Lab 8.0 software (for statistics see Supplementary data). The data were gathered from at least three separate experiments involving up to five concentrations in the range 10–100 nM. Comparison of EC_50_ values for gapmers between WT and Mutant target RNA alleles allowed to evaluate allele-preference for cleavage of tested gapmers. Two last columns of the table present WT/Mut ratio of gapmer selectivity and the selectivity of mismatched gapmers relative to complementary gapmer fKM.

In the G-to-C case, the starting point for gapmer design was the single G-dG mismatch in the WT RNA/ASO duplex, placed not in the DNA gap center but closer to the 3′-end (position 5 of the 13 nucleotide-long gapmer). The reason for this different localization was to minimize the number of repeats in the target sequence. We realized that such placement is not in the cleavage site and expected it would have a rather negligible influence on the yield of cleavage by RNase HI. However, this single G-dG mismatch turned out to limit the cleavage efficiency of WT RNA by approximately 50% and Mut RNA only by approximately 15%, without a change in cleavage pattern (fKM gapmer, Figure S2B). This result was encouraging, and therefore for the *SCA3* target many different options of double mismatches, including the G-dG mismatch and tandem mismatches, were tested *in vitro* with RNase HI assays (Fig. 10). Several gapmers presented interesting allele-dependent cleavage efficiency, significantly differentiating between WT and Mut RNA cleavage by RNase HI. The effect of the thermodynamic destabilization by the G-dG mismatch was significant and independent of the presence of additional mismatches (Figure S3A). The results from *HeLa* cells indicated that a single mismatch in this case is not sufficient to diversify the degradation efficiency of alleles. The mutant-complementary gapmer fKM reduced the levels of both alleles to similar values, no matter the gapmer concentration. The most efficient preference for mutant allele cleavage was observed with the f5 gapmer (Figs 10 and S3B), where the G-dG mismatch was supported by a tandem mismatch at the 5′-end of the DNA gap that is at the site of RNA cleavage. The highest statistically significant difference in WT/Mut allele ratio was reached with a concentration of approximately 100 nM gapmer, at which the WT RNA level was decreased by approximately 25% and Mut RNA level slightly over 50%. Gapmer f5 indicated some selectivity toward the mutant allele *in vitro* (Fig. 10B), but in comparison to other gapmers, it presented lower activity generally, and therefore it was not expected to give good results. Ultimately, only the f5 gapmer preferentially reduced the level of the mutant allele in *HeLa* cells (Fig. 10C).

### Allele-preferential RNA cleavage in presence of bulges

Formation of bulges in RNA/ASO duplexes was accomplished by changing the length of ASOs by one nucleotide relative to the 13-nucleotide target RNA. Consequently, single nucleotide bulges occurred in gapmer or RNA strands and were placed at SNP or next to SNP sites. The RNase H assay for the *APP*, *SOD1* and *SCA3* target RNAs revealed that the analyzed bulges had no influence on the cleavage yield of the two alleles by RNase HI (Figure S4), but they significantly changed the pattern of target RNA cleavage (Figure S5A). This observation confirms that interactions occurring between nucleotides of base pairs in the region of RNA cleavage determine whether the RNA can be a substrate for RNase H.

## Conclusions

Complementary base pairing is a principal assumption for antisense oligonucleotide activity, including ASO and siRNA. Every mismatch within the duplex formed with target RNA diminishes target specificity and presumably increases the number of potential off-target interactions. During *in vitro* experiments with recombinant *E*. *coli* RNase HI, we observed, that even if full complementarity of ASO/target RNA duplexes is maintained, different RNAs are cleaved by RNase HI with different efficiency. For example, complementary duplexes of *APP*-717 RNA variants were cleaved 2–3 times slower than mismatched duplexes and complementary duplexes of other targets. The introduction of a single A-dA mismatch to these duplexes at the 3′-end of the DNA gap significantly improved cleavage efficiency of both RNA 717 alleles by RNase HI (Fig. 5B, d8 and d9 gapmers). *SOD1* RNA target was also an example confirming that mismatches may enhance cleavage by RNase H. In the case of *SOD1* RNA, we observed better cleavage of the WT RNA 4 variant, which in duplexes with different ASOs always carried one mismatch more than the Mut RNA (Fig. 9B). The increased number of mismatches in *SOD1* RNA/ASO duplexes mostly did not affect the cleavage unless the mismatch occurred in the cleavage site.

Often, the presence of mismatches caused a change of the major cleavage sites into minor ones or conversely, shifted them by one or two nucleotides (Figure S5). The shifts of the cleavage sites occurred between the eighth and the twelfth nucleotides from the 5′-end of the RNA, concerned all analyzed SNP cases and were dependent on the arrangement of the mismatches within the duplex. As a result of mismatches, for several gapmer/RNA duplexes (i.e., f13 or f14 gapmers and *SCA3* RNAs) shifts of the RNA cleavage site out of the DNA gap region of the duplex were observed to occur opposite from the LNA and 2′-O-methylRNA nucleotides of the ASO. The sugar residues of these nucleotides adopt a different conformation (*C2′-endo*) that limits RNase H cleavage activity. However, in the cases described above, despite the fact that RNA cleavage occurred in this unexpected site within duplexes, it did not significantly influence cleavage efficiency.

Although it is possible to control the influence of each variable in the experiment *in vitro*, there is no such possibility in cells. Many factors including accessibility of the hybridization site, the number of potentially existing sequence-dependent and independent off-targets, the actual concentration of antisense oligonucleotide due to differences in transfection efficiency, the condition of the cells and the phase of cell cycle during transfection determine the final effect that is observed. Therefore, the consistency between cleavage results in *in vitro* assays and RNA profiles from *HeLa* cells seemed interesting. Several sets of control experiments were designed to validate gapmers activity in cells. Firstly, GFP levels in *HeLa* cells were measured, to which empty plasmid (without an insert) was co-transfected with the gapmer. Often we observed a decrease in GFP expression, despite gapmers’ target was absent in the cell (Figure S6). Secondly, random DNA oligonucleotide R434 (5′ TCATGTCAGACGTTCTGCCCTC, 22nt) in 50 nM concentration, instead of gapmer, was co-transfected into cells with the WT or Mut plasmid. The effect of R434 was not SNP-preferential relative to APP WT and Mut (Flemish variant) target, however we observed significant decrease of GFP level in case of plasmid without insert (Figure S7), similarly as it was in the previously mentioned control experiment. Moreover, we designed a cross-targeting control experiment, in which we checked if the gapmer designed to target APP construct (Mutant-complementary dKM) influences the expression of SOD1 and SNCA constructs, or the gapmer designed to target SOD1 construct (Mutant-complementary aKM) influences the expression of APP and SNCA constructs, or whether the gapmer designed to target SNCA (Mutant-complementary kKM) influences the SOD1 and APP targets. These experiments showed that APP and SNCA targets were insusceptible to the presence of gapmers designed to target other RNAs (Figure S8 and S9, respectively), and it was additionally confirmed by statistical insignificance of the changes compared to the control (cells transfected with WT construct only). However, in case of SOD1 target, we observed statistically significant decrease of GFP level in the presence of gapmers designed to target APP and SNCA (Figure S10).

All these controls showed that antisense gapmers definitely reach their RNA targets in cells. However, in the absence of the target, ASO gapmers as well as random DNA oligonucleotide interact nonspecifically, decreasing the level of GFP RNA (empty plasmid without target insert). Certainly, these nonspecific effects also occur in the presence of the target, diminishing the pool of ASO that might successfully bind. In case of allele-preferential RNA cleavage based on SNPs, the concentration of ASO and proper mismatch localization are equally important. When concentration of ASO is too low, the gapmer might not reach its target site, and when it is too high, it acts mostly nonspecifically. For SNP-selectivity of ASO, its concentration is the most important parameter to determine, because the behavior is similar to competitive inhibition, where stoichiometry plays crucial role. Dose-response curves of ASO activity in *HeLa* cells enabled us to clearly illustrate these dependencies for the most efficient oligonucleotides.

Mismatches form non-canonical base pairs that vary in strength and may significantly influence duplex stability and structure^67,68^. Their presence results in changes to the local duplex structure that may be beneficial for differentiating cleavage of the target RNA alleles by RNase H. We observed this effect *in vitro* in RNase HI assays and successfully used it to selectively degrade Mut RNAs in some SNPs. The approach worked best with C-to-G transversion in the context of the *APP* RNA target, for which two gapmers were found that caused preferential degradation of mutant RNA by RNase H1 *in HeLa* cells. Their manner of action is based on proper localization of a defined set of mismatches within the RNA/ASO heteroduplex.

The general conclusion for all analyzed SNP types is that the effect of preferential degradation of specific RNA alleles is strongly dependent on the ASO concentration. Comparison of dose–response curves of ASO gapmers illustrates this dependency. For ASO, which can cause SNP-preferential RNA cleavage relative to concentration, dose-response curves for the levels of two RNA alleles did not overlap. The more allele-preferential properties gapmer demonstrated, the clearer this relationship was. To determine the overall SNP-preference of specific ASOs, we compared EC_50_ values for WT and Mut RNAs. Three ASOs which were characterized by a significantly lower EC_50_ value in case of Mut RNA than WT RNA (b8, b8-a and h2) will be further evaluated, also in different cell line. In most cases, mismatches that appear as a consequence of SNP change do not drastically alter the efficiency of RNA degradation, and the observed mutual deviation of the curves for both alleles is small.

Thermodynamic data concerning RNA/RNA as well as RNA/DNA short duplexes indicate that the central position of the mismatch causes the highest impairment of duplex stability and structure^55,69,70^. For RNase H degradation efficiency, the most important factor is the cleavage region of the RNA/ASO duplex, which depends mostly on duplex length and structure. Therefore, selective mutant RNA cleavage using gapmers depends on ASO length, and the type, number and position of mismatches, which must be determined on the basis of target sequence. Our studies revealed that in the case of maintaining allele-preference of RNase H cleavage, elongation of the DNA gap is more favorable than elongation of the modified wings of the gapmer. Although it is extremely difficult to design a universal gapmer template for selective degradation of SNP-mutants at the RNA level, the knowledge of properties of different types of mismatches will be very useful to design antisense oligonucleotides that support allele-selective RNA cleavage with ribonuclease H.

## Methods

### Oligonucleotide synthesis

Short RNA oligonucleotides (13-, 15-, and 20-mers), ASO gapmers (13-, 15-, 17-, and 20-mers) and primers for PCR reactions were synthesized on a BioAutomation MerMade12 DNA/RNA synthesizer using β-cyanoethyl phosphoramidite chemistry^71^, and commercially available phosphoramidites (ChemGenes, GenePharma). The details of oligoribonucleotide deprotection and purification are described previously^72,73^.

### UV melting experiments

Oligonucleotides were melted in buffer containing 100 mM NaCl, 20 mM sodium cacodylate and 0.5 mM Na_2_EDTA, at pH 7. Only in the case of the *SCA3* G-to-C SNP, buffer containing 10 mM NaCl was used, because of the inability to calculate thermodynamic parameters for 100 mM NaCl. Single-strand oligonucleotide concentrations were calculated from the absorbance above 80 °C, and single strand extinction coefficients were approximated using a nearest-neighbor model^74,75^. Absorbance vs. temperature melting curves were measured at 260 nm with a heating rate of 1 °C/min in the range of 2 to 90 °C on a Beckman DU 640 or JASCO V-650 spectrophotometer with a thermoprogrammer. Melting curves were analyzed, and thermodynamic parameters were calculated from a two-state model with the program MeltWin 3.5^76^. Statistical analysis of thermodynamic data was carried out using MeltWin 3.5 software. The methods for determining sampling errors in ∆G°_37_, ∆H° and ∆S° from the linear regression of T_M_^−1^ vs. ln(C_T_/4) plots using standard statistical analysis has been previously described^77^. Based on results obtained for nine concentrations of each duplex, T_M_^−1^ vs. ln(C_T_/4) dependence was determined, from which the calculated ∆G°_37_ values for averaged 10^–4^ M concentrations were compared with that derived from averaging the fits to individual melting curves. Differences below 15% indicated that a two-state model is reasonable for melting of the duplex analyzed.

### RNase H assay

Screening of antisense gapmers was carried out using an RNase H assay. RNAs were labeled at 5′-end with γ^32^P–ATP using T4 polynucleotide kinase (long RNAs) or with fluorescein (FAM) during oligonucleotide chemical synthesis (short RNAs). Labeled RNA was mixed with ASO in H-buffer (20 mM Tris-HCl pH 7.8, 40 mM KCl, 8 mM MgCl_2_, 1 mM DTT) at proportions optimized in a separate experiment (10:1 for RNA/gapmers). The mixture was denatured for 3 min at 80 °C and renatured at room temperature for 5 min to form duplexes. Next, 0.4 U of RNase H (*E*. *coli*) was added, and the mixture was incubated for 20 min in 37 °C. The time of RNase H digestion, as well as the enzyme concentration, was optimized according to the fully complementary RNA/gapmer duplexes. Experiments were done with recombinant *E*. *coli* RNase H because of its wide commercial availability. Cleavage products were separated on a 16% polyacrylamide gel with 7 M urea and visualized by autoradiography. Digestion efficiency was evaluated from gel images using the MultiGauge 3.0 program (Fuji). The same assay was used to determine the kinetics of RNase H cleavage. A mixture of the appropriate duplex in H-buffer with ribonuclease H was incubated at 37 °C, and 10 μL aliquot samples were collected after 30 sec, 1, 5, 10, and 20 min of reaction. In most cases, 20 min was sufficient for complete RNA hydrolysis. The assay was performed at least three times for each tested oligonucleotide. Mean ± SD of digestion efficiency was evaluated with Microsoft Excel software.

### HeLa cell line assays and RT-qPCR analysis

*HeLa* cell line experiments were performed using the 99-nucleotide fragment of the gene of interest (in Wild-type or Mut variant) inserted into a pEGFP expression plasmid upstream of the GFP protein. One day before transfection, *HeLa* cells were passaged to 24-well plates and cultured in a standard RPMI 1640 medium containing 10% FBS (Invitrogen), 1x Antibiotic-Antimycotic solution (Sigma Aldrich) and 1x MEM vitamins solution (Sigma Aldrich). When the confluence reached ca. 90% per well, co-transfection of the pEGFP plasmid constructs (Wild-type or Mut) at a concentration of 1 μM and gapmers at concentrations of 0, 10, 25, 50, 75 and 100 nM was carried out with Lipofectamine 2000 (Invitrogen) in medium without antibiotics. For certain gapmers, 200 and 300 nM concentrations were also tested. After 24 h incubation, cells were washed with phosphate-buffered saline (PBS) and viewed under a fluorescence microscope, and then total RNA was extracted. Each transfection experiment (using different concentrations of various ASOs) was repeated at least three times. For quantitative analysis of silencing, total RNA from cells was isolated using the TRIzol method^78^ as described previously^79^. Next, 0.5 μg of RNA was subjected to DNase I (Life Technologies) treatment. RNA quality was controlled by separation on 1.5% agarose gels. cDNA, which was the template for qPCR, was obtained from a reverse transcription reaction using iScript cDNA Synthesis Kit (Bio-rad). qPCR was performed on a CFX96 real-time PCR system (Bio-Rad) using iTaq SYBR Green Supermix (Bio-rad) and 96-well clear plates. The level of GFP mRNA was quantified with the use of target gene primers: GFPf 5′-GCTGACCCTGAAGTTCATC, GFPr 5′-GCTCCTGGACGTAGCCTTC, and normalized to human β-actin levels (reference gene primers: ACTf 5′-AGGCACCAGGGCGTGATG, ACTr 5′-TGATCTGGGTCATCTTCTCGC). The qPCR cycles were as follows: 95 °C for 5 min; (95 °C for 10 sec and 60 °C for 1 min) for 40 cycles. Each qPCR experiment was repeated at least twice.

### Determining the EC_50_ of a gapmer oligonucleotide

Five different concentrations ranging from 0–100 nM (mostly 10, 25, 50, 75, 100 nM) were examined for each ASO transfected to *HeLa* cells. Semi-log plots of dose-response dependency were designated for the oligonucleotides in OriginLab 8.0 software, and they served as estimations of the EC_50_ values of each antisense oligonucleotide – RNA variant pair. Comparison of EC_50_ values for wild type and mutant RNA variants allowed to evaluate allele-preference of antisense oligonucleotides.

### qPCR statistical analysis

Statistical analysis of the qPCR results was performed with Bio-rad CFX Manager 3.0 and OriginLab 8.0 software. Antisense oligonucleotides were tested in various concentration sets, and transfection of each set was repeated two to five times (biological replicates). Quantitative PCR for each set was repeated at least twice (technical replicates). Standard curves for target and reference genes were determined to control for PCR efficiency of each reaction, which ranged from 91 to 97%. The results from technical repeats were gathered for each concentration set transfected with WT or Mut constructs to determine mean relative expression and standard deviation (Bio-rad CFX Manager 3.0). Samples for which the obtained Cq value differed by more than 0.5 from the others were excluded from the analysis. Next, using one-way ANOVA analysis (OriginLab 8.0) the normalized relative expression of GFP (which was fused with WT or Mut RNA alleles of the analyzed genes) from biological replicates for WT and Mutant was compared at a significance level of 0.05 for each of the conditions analyzed. Statistically significant differences in mean expression between WT and Mut alleles (P < 0.05) were observed only at certain concentrations. (Supplementary data file 2).

### Data availability

All data generated or analysed during this study are included in this published article (and its Supplementary Information files).

## Electronic supplementary material

Dataset 1

Dataset 2

Dataset 3
