# Supplementary material for: Influence of mismatched and bulged nucleotides on SNP-preferential RNase H cleavage of RNA-antisense gapmer heteroduplexes
Source: Sci Rep. 2017 Oct 2;7:12532. doi: 10.1038/s41598-017-12844-z (PMC5624880; doi:10.1038/s41598-017-12844-z)
Supplement: Supplementary file 1 — Dataset 1 [file 41598_2017_12844_MOESM1_ESM.doc]

Supplementary materials

**Influence of mismatched and bulged nucleotides on SNP-preferential RNase H cleavage of RNA-antisense gapmer heteroduplexes**

# Dorota Magner, Ewa Biala, Jolanta Lisowiec-Wachnicka and Ryszard Kierzek

Institute of Bioorganic Chemistry Polish Academy of Sciences, 61-704 Poznan, Noskowskiego 12/14, Poland

Correspondence should be addressed to D. Magner or R. Kierzek, Telephone (+48) 61-852-8503, Fax (+48) 61-852-0532, E-mail: dorotaw@ibch.poznan.pl, rkierzek@ibch.poznan.pl,


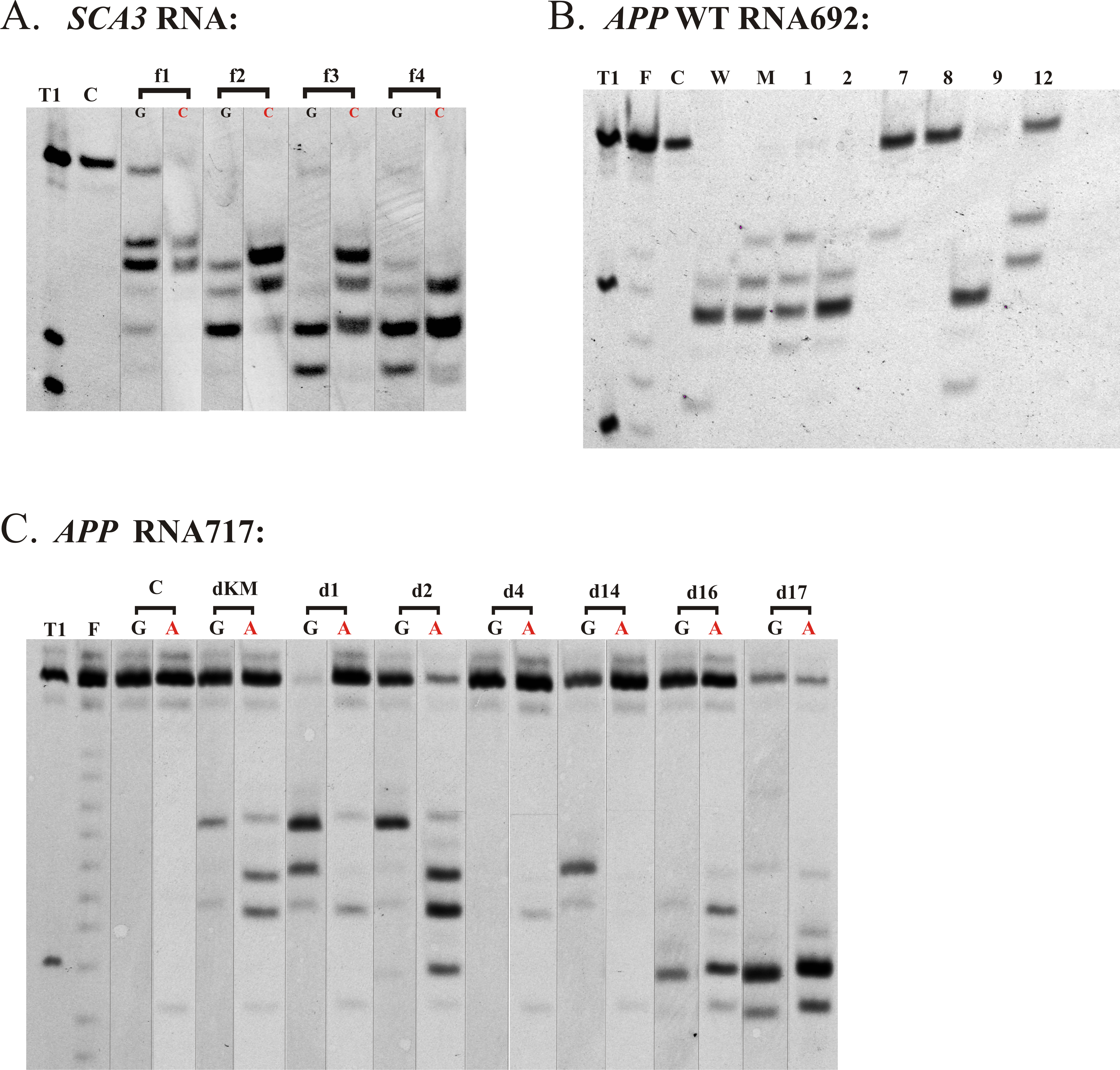


**Fig. S1. Results of RNase H cleavage for different RNA targets, showing different cleavage yields and patterns relative to mismatches content. A.** ASO gapmers (f1-f4) introducing bulges to RNA/gapmer heteroduplex did not differentiate the yield of SCA3 WT (G allele) and Mut (C allele) RNA cleavage by RNase H but changed the pattern of cleavage, T1- RNase T1 ladder, C – Control - WT RNA cleaved in absence of ASO gapmer. **B.** RNase H cleavage of APP WT RNA in presence of gapmer carrying different mismatches arrangements: W – wild type complementary bKW gapmer, M – mutant complementary bKM gapmer ( C-dC mismatch at position 5/13 from 5’ end of the RNA), 1 – b1 gapmer (tandem GC-dGdC mismatch at positions 4-5/13 from 5’ end of the RNA), 2 – b2 gapmer (G-dG mismatch at position 4/13 from 5’ end of the RNA), 7 – b7 gapmer (quadruple mismatch CAGA-dCdAdGdA at positions 5-8/13 (internal loop) from 5’ end of the RNA), 8 – b8 gapmer (triple mismatch C-dC at position 5/13 and AG-dAdG at positions 9-10/13 from 5’ end of the RNA), 9- b9 gapmer (tandem mismatch AG-dAdG at positions 9-10/13 from 5’ end of the RNA), 12 – b12 gapmer (triple mismatch CAG-dCdAdG at positions 5-7 from 5’ end of the RNA), T1 – RNase T1 ladder, F – WT RNA alkaline hydrolysis, C – Control - WT RNA cleaved in absence of ASO gapmer **C.** ASO gapmers differentiating RNase H cleavage and pattern of APP-717 WT (G allele) and Mut (A allele) RNA: T1- RNase T1 ladder of WT RNA, F – WT RNA alkaline hydrolysis, C - Control - WT (G) and Mut (A) RNA cleaved in absence of ASO gapmer, dKM-d17 – ASO gapmers. Lanes on gels (A) and (C) were reorganized to directly compare WT and Mut cleavage yields in presence of particular ASO. Original images representing (A) and (C) gels are included as Fig. S15 and S16, respectively.

A. SOD1 termodynamics

B. SOD1 dose-response curves for selected ASO gapmers

**aKM**

**a13**

**a14**

**Fig. S2. (A) Results of thermodynamic measurements of SOD1 RNA variants –antisense gapmers heteroduplexes.** Oligonucleotides, which differentiated two alleles cleavage yields in RNase H assay were only measured. Parameters for more stable duplex of the two (wild type/ASO or mutant/ASO) are bolded. **(B) Dose-response curves for SOD1 targeting, selected ASO gapmers.**

A. SCA3 termodynamics

B. SCA3 dose-response curves for selected ASO gapmers

**fKM**

**f5**

**Fig. S3. (A) Results of thermodynamic measurements of SCA3 RNA variants –antisense gapmers heteroduplexes.** Oligonucleotides, which differentiated two alleles cleavage yields in RNase H assay were only measured. Parameters for more stable duplex of the two (wild type/ASO or mutant/ASO) are bolded **. (B) Dose-response curves for SCA3 targeting, selected ASO gapmers.**

**Fig. S4. Results of RNase H *in vitro* assay for antisense gapmers introducing bulges to RNA/ASO duplex. Gapmers of 12 and 14 nucleotides in length were designed to 13 nt-long RNAs.** A. SCA3 target; B. SOD1 target; C. APP-692 target; D**.** APP-717 target.

**
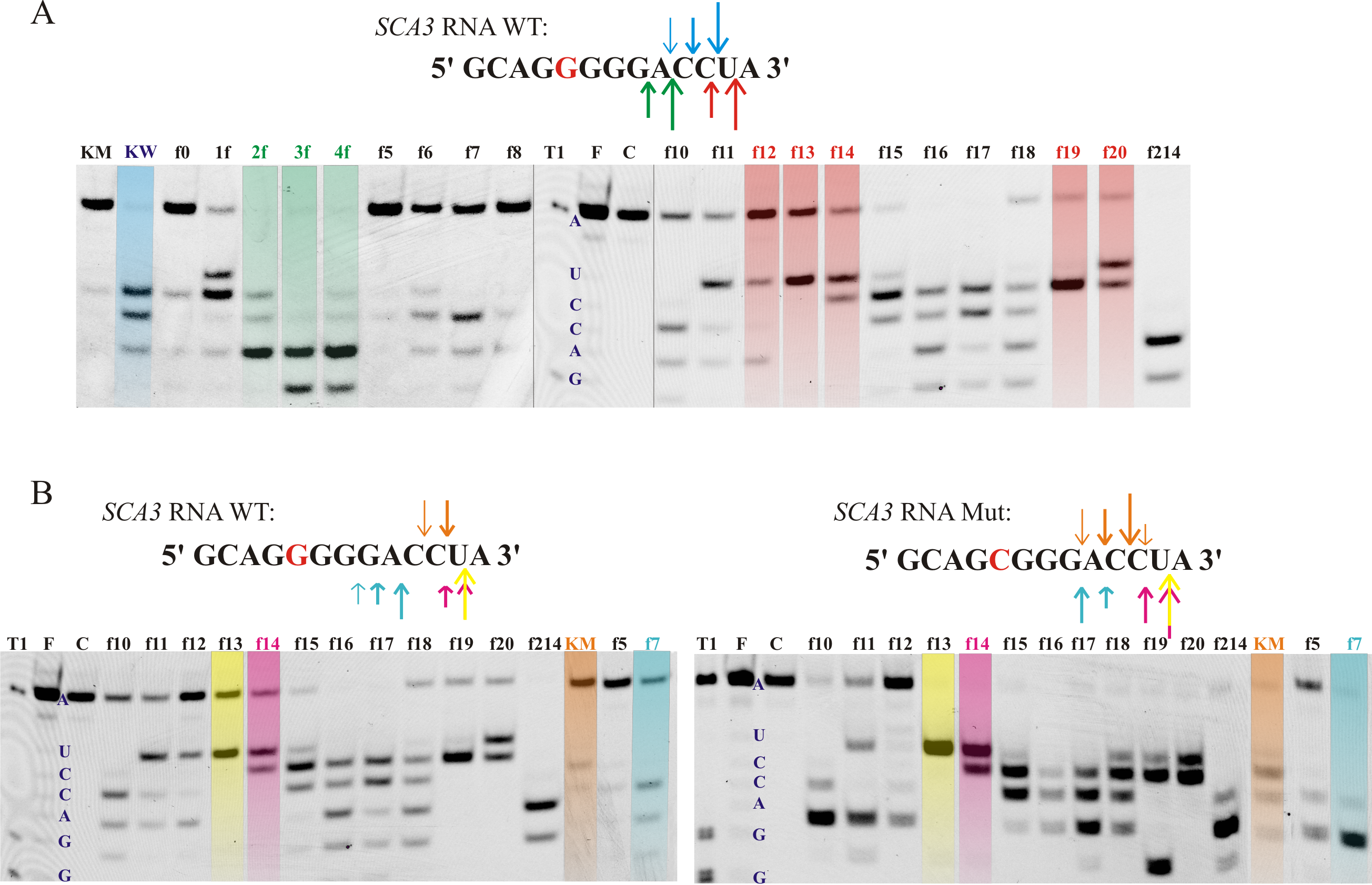
**

**Fig. S5. Sites of cleavage of SCA3 RNA alleles relative to presence of different gapmers.** Colors of arrows corresponds to colors of gel lanes presenting RNase H yield and pattern of RNA cleavage in presence of particular gapmer; **(A)** RNase H cleavage patterns of WT SCA3 RNA in presence of most tested gapmers: blue – fully complementary fKW gapmer, green – gapmers introducing bulges, red – gapmers which significantly change cleavage pattern. **(B)** Comparison of WT and Mut RNA cleavage yields and patterns in presence of tested gapmers;

T1- RNase T1 ladder, F – RNA alkaline hydrolysis C – Control – RNA cleaved in absence of ASO gapmer, KW- fKW (wild type complementary gapmer), KM – fKM (mutant complementary gapmer), f0-f214 ASO gapmers introducing different mismatches arrangements, 1f-4f – ASO gapmers introducing bulges.

A.


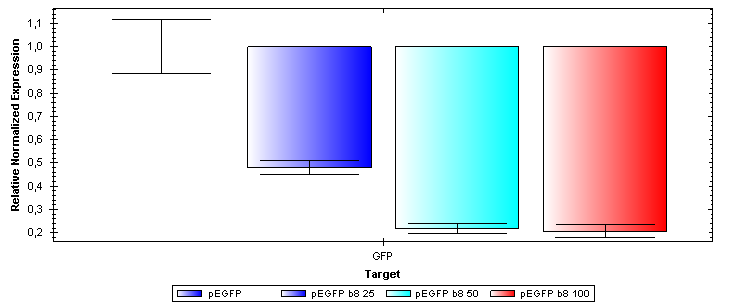


B.


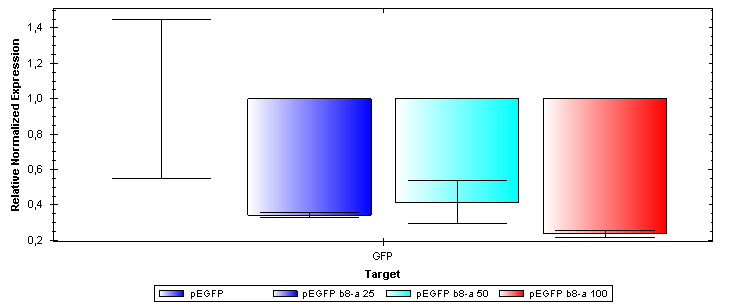


C.


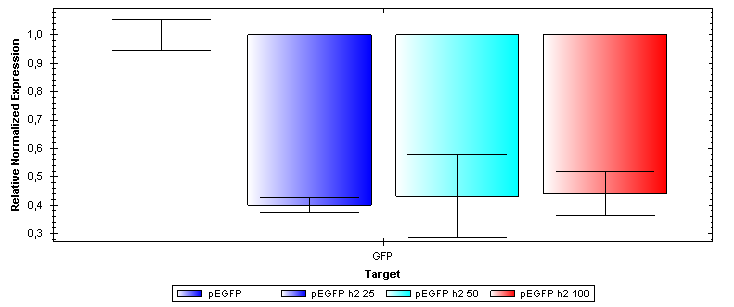


**Fig. S6. Results of control experiment, presenting activity of selected gapmers relative to empty pEGFP plasmid (without insert including target).** A – b8 gapmer in concentration of 25, 50 and 100 nM, B – b8-a gapmer in concentration of 25, 50 and 100 nM, C – h2 gapmer in concentration of 25, 50 and 100 nM

A. APP WT-pEGFP construct


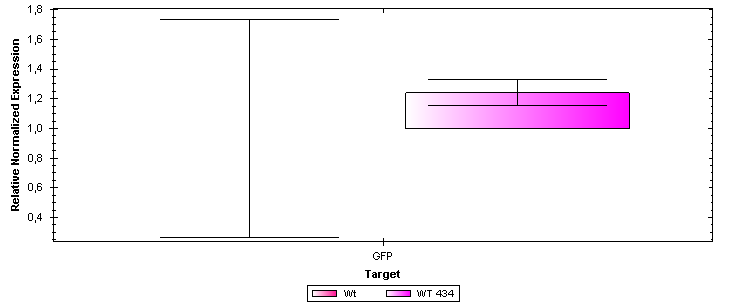


B. APP Mut-pEGFP construct (Flemish variant)


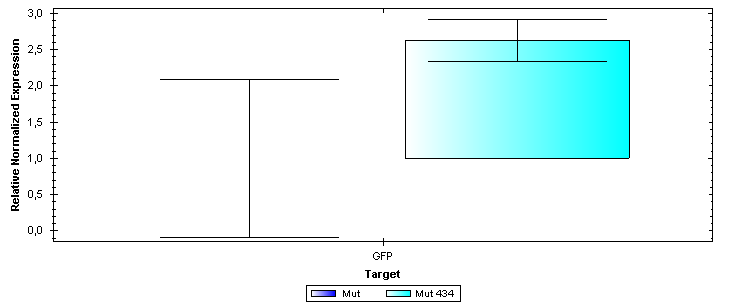


C. pEGFP plasmid without insert


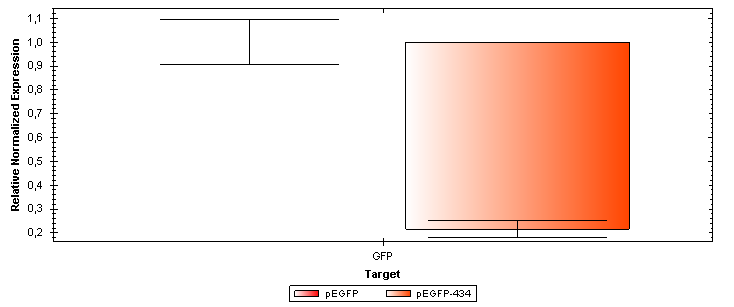


**Fig. S7. Expression of GFP construct in presence of control, random DNA oligonucleotide R434** (5’TCA TGT CAG ACG TTC TGC CCTC - 22nt) in 50 nM concentration; A – WT APP construct, B – Mut APP construct, C- pEGFP plasmid without insert


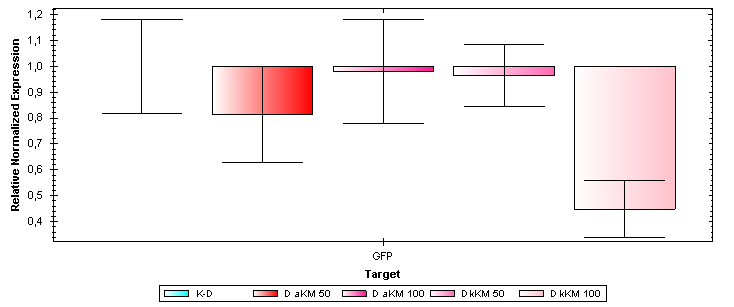


**Fig S8. APP Cross-target control: antisense gapmers designed to SOD1 target (aKM) and SNCA target (kKM) showed no significant influence on wild-type APP-pEGFP construct.**

**K-D** – WT APP construct, **D aKM 50** – WT APP construct in presence of 50 nM aKM gapmer, **D daKM 100** - WT APP construct in presence of 100 nM aKM gapmer, **D kKM 50** - WT APP construct in presence of 50 nM kKM gapmer, **D kKM 100** - WT APP construct in presence of 100 nM kKM gapmer.


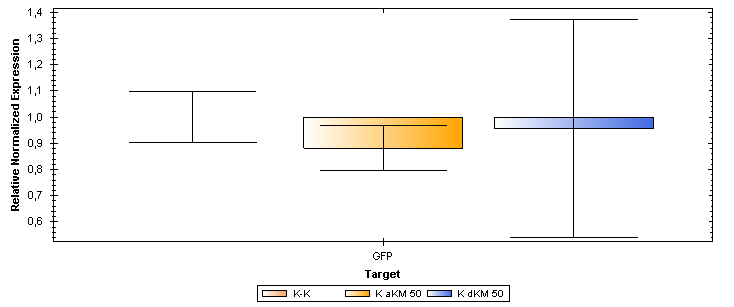


**Fig S9. SNCA Cross-target control: antisense gapmers designed to SOD1 target (aKM) and APP target (dKM) showed no significant influence on wild-type SNCA-pEGFP construct.**

**K-K** – WT SNCA construct, **K aKM 50** – WT SNCA construct in presence of 50 nM aKM gapmer (SOD1 targeted), **K dKM 500** - WT SNCA construct in presence of 50 nM dKM gapmer (APP targeted).


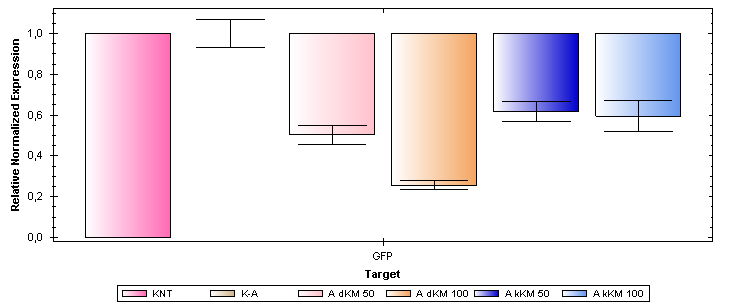


**Fig S10. SOD1 Cross-target control: antisense gapmers designed to APP target (dKM) and SNCA target (kKM) decreased expression of wild-type SOD1-pEGFP construct**.

**KNT**- non-transfected control, **K-A** – WT SOD1 construct, **A dKM 50** – WT SOD1 construct in presence of 50 nM dKM gapmer, **A dKM 100** - WT SOD1 construct in presence of 100 nM dKM gapmer, **A kKM 50** - WT SOD1 construct in presence of 50 nM kKM gapmer, **A kKM 100** - WT SOD1 construct in presence of 100 nM kKM gapmer.

A. GFP standard curve


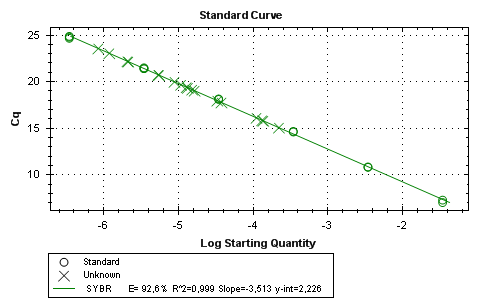


B. ACTIN standard curve


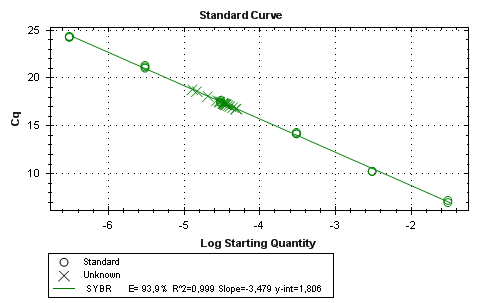


C. GFP/ACTIN superposition


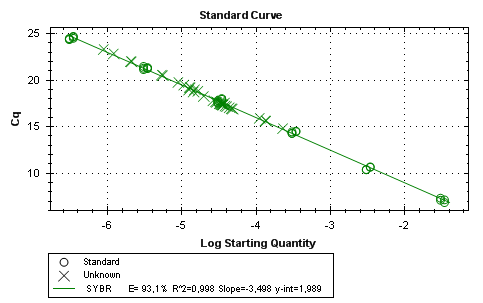


**Fig. S11. Normalization of qPCR.** Examples of standard curves for GFP (target, chart A), β-actin (reference, chart B), and their superposition for relative gene expression normalization (chart C). Parameters of the curves were determined with Bio-Rad CFX Manager 3.0 software. As the standard curves for target and reference genes run in parallel, expression changes were determined with direct method based of comparison of the normalized target expression with the control sample, which usually was transfection of plasmid constructs only.

A. APP standard curve


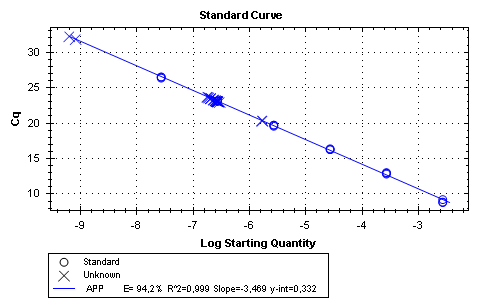


B. ACTIN standard curve


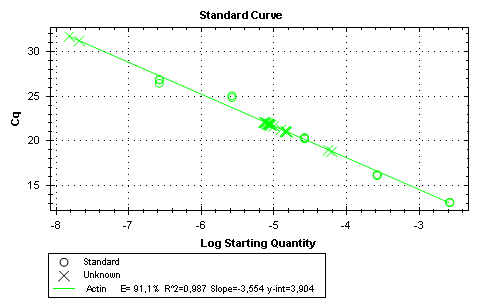


C. APP/ACTIN superposition


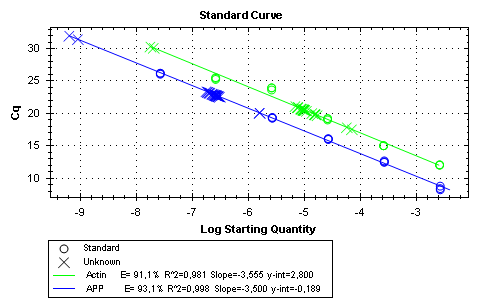


**Fig. S12. Normalization of qPCR.** Examples of standard curves for APP (target, chart A), β-actin (reference, chart B), and their superposition for relative gene expression normalization (chart C). APP level was measured as a reference in control experiment. Parameters of the curves were determined with Bio-Rad CFX Manager 3.0 software. As the standard curves for target and reference genes run in parallel, expression changes were determined with direct method based of comparison of the normalized target expression with the control sample, which usually was transfection of plasmid constructs only.

A.


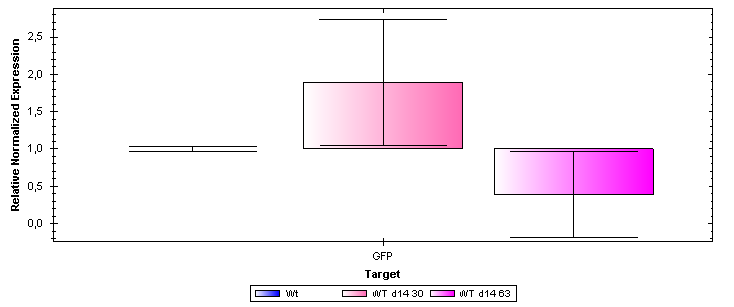


B.


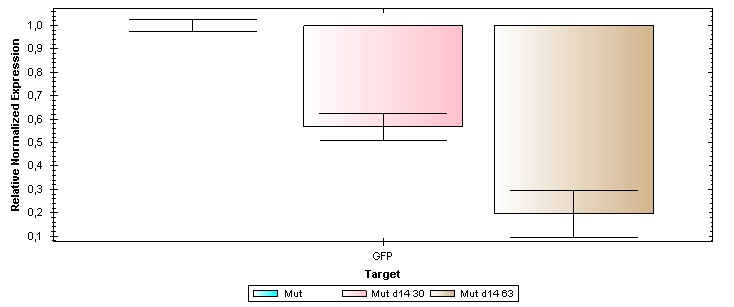


**Fig. S13. Validation of dose-response curve models determined for d14 gapmer.** Two concentrations of d14 (EC_50_ for wild type – 63 nM, and EC_50_ for Mut – 30 nM) were tested in co-transfection with WT/Mut pEGFP constructs and their allele-preferential effect was confirmed. A – Wild type APP construct expression, B – Mut APP (London variant) construct expression.


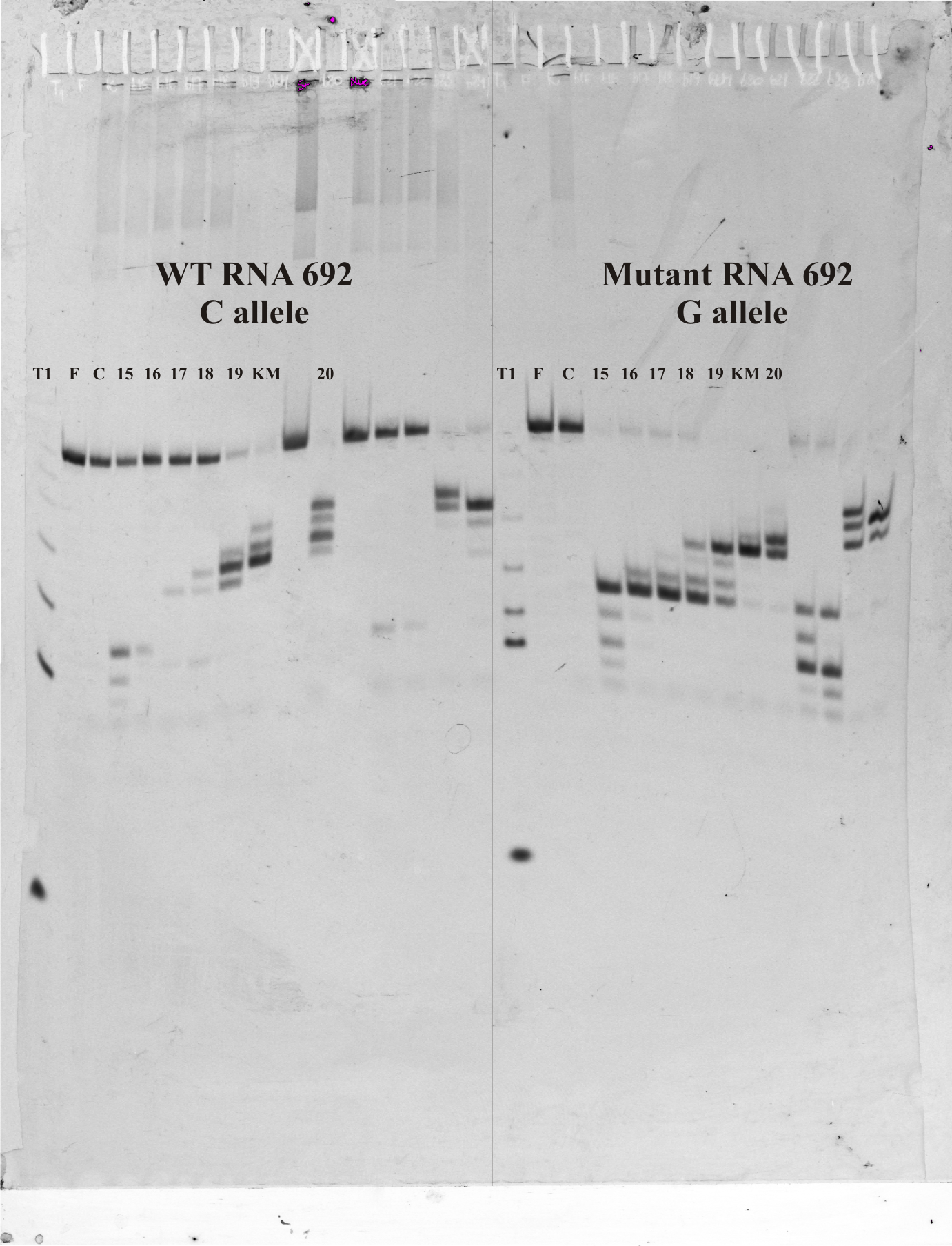


**Fig. S14.** **Original gel image for figure 2.** In figure 2, lanes of interest (numbered 15-20) were cropped and rearranged, to skip not applicable data (lanes not numbered) and for clearer comparison of RNase H cleavage yields of wild type and Mut RNAs in presence of gapmers of interest.


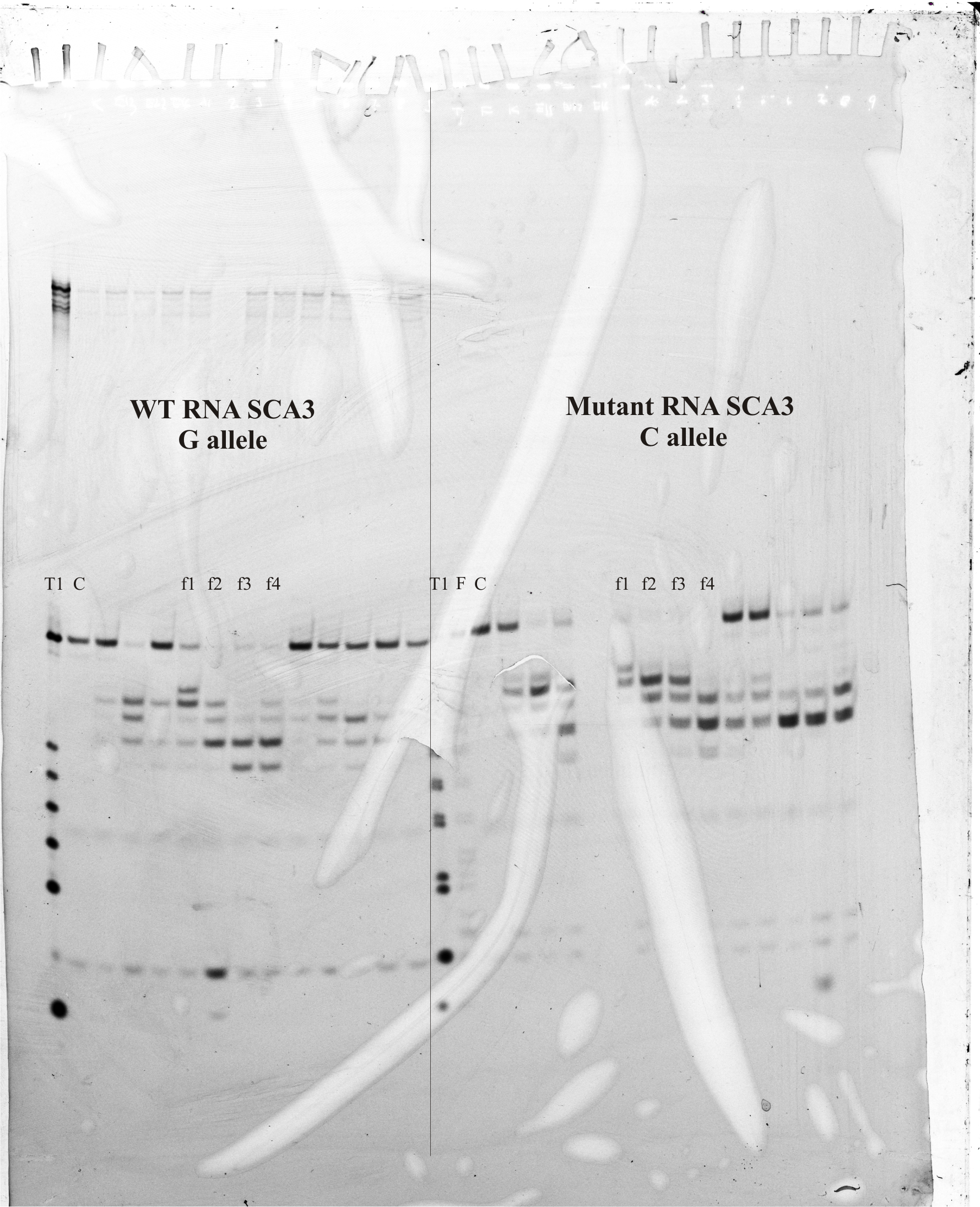


**Fig. S15. Original gel image for figure S1A.** In figure S1A lanes of interest (numbered f1-f4) were cropped and rearranged for clearer comparison of RNase H cleavage yields of wild type and Mut RNAs.


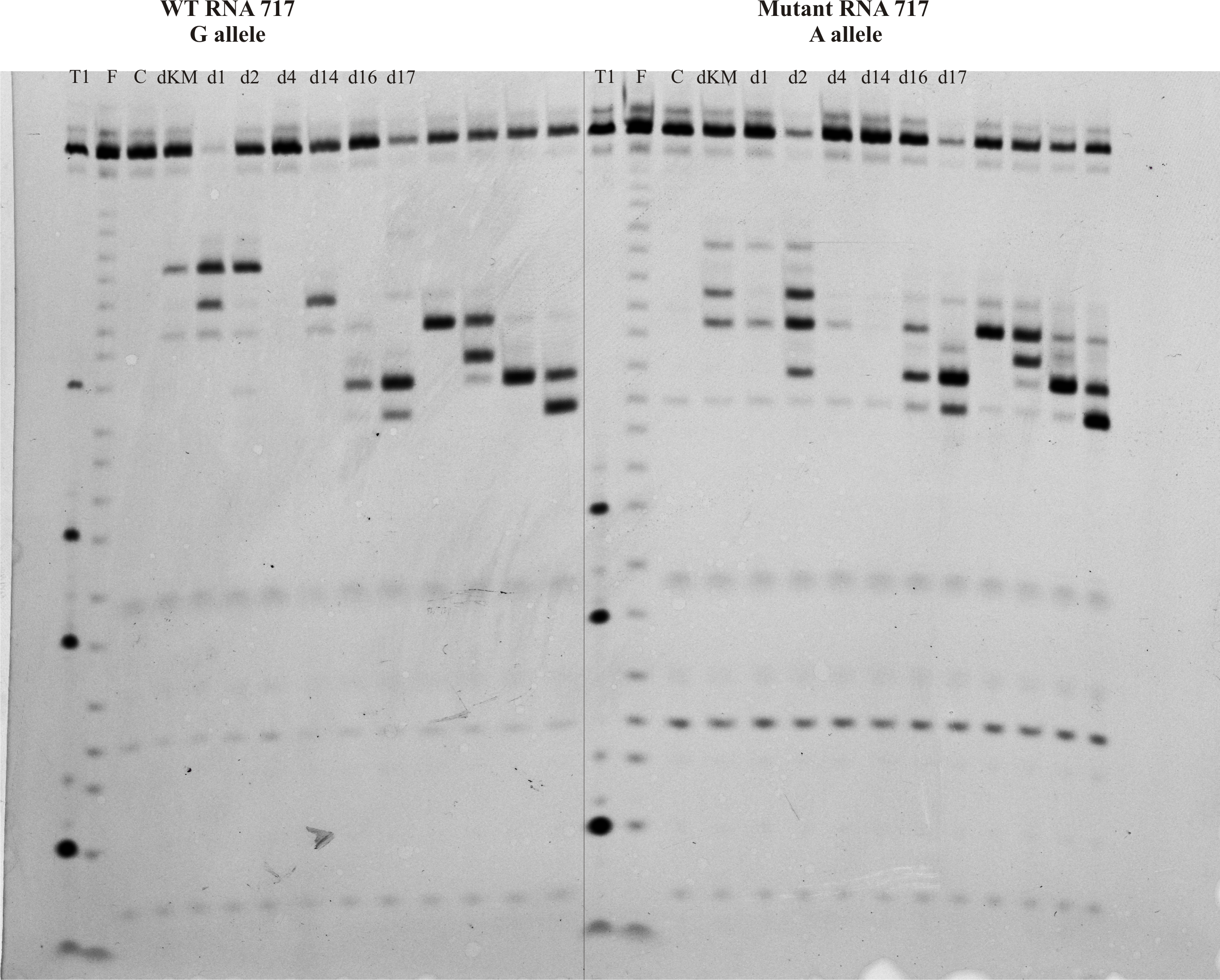


**Fig. S16. Original gel image for figure S1C.** In figure S1C lanes of interest (numbered dKM-d17) were cropped and rearranged for clearer comparison of RNase H cleavage yields of wild type and Mut RNAs.
